# RAGE-Mediated Signalling in Gynaecological Disorders: Review of Molecular Mechanisms and Therapeutic Perspectives

**DOI:** 10.1017/erm.2026.10055

**Published:** 2026-06-15

**Authors:** Krzysztof Łuszczyński, Magdalena Dec, Aleksander Chodowiec, Marcin Radziszewski, Robert Zdanowski, Monika Szafarowska, Paweł Kamiński, Paweł Włodarski, Anna Lutyńska, Aneta Ścieżyńska

**Affiliations:** 1 Laboratory of Molecular Oncology and Innovative Therapies, Military Institute of Medicine National Research Institute, 128 Szaserów Street, 04-141 Warsaw, Poland; 2 Department of Histology and Embryology, Medical University of Warsaw, 02-004 Warsaw, Poland; 3 Department of Thoracic Surgery, National Medical Institute of the Ministry of the Interior and Administration, 02-507 Warsaw, Poland; 4 Department of Gynecology and Oncological Gynecology, Military Institute of Medicine National Research Institute, 128 Szaserów Street, 04-141 Warsaw, Poland

**Keywords:** AGEs, cancer, glycation, gynaecology, inflammation, pregnancy, RAGE, targeted therapy

## Abstract

**Background:**

The receptor for advanced glycation end-products (RAGE) is a unique multi-ligand member of the immunoglobulin superfamily that exists in both membrane-bound and soluble forms. Under physiological conditions, RAGE expression is low in most tissues; however, it is markedly upregulated in response to tissue injury, inflammation or metabolic stress. Ligand-induced activation of RAGE initiates complex intracellular signalling cascades that regulate inflammation, extracellular matrix remodelling, cell proliferation, survival and migration.

**Methods:**

While the contribution of RAGE to diabetes and chronic inflammatory diseases is well established, its role in gynaecological disorders remains insufficiently characterized.

**Results:**

This comprehensive review summarizes current evidence on the involvement of RAGE in the pathogenesis of benign gynaecological disorders, such as endometriosis and polycystic ovary syndrome (PCOS), pregnancy-related complications and malignant neoplasms of the female reproductive tract.

**Conclusions:**

It also discusses emerging therapeutic strategies aimed at targeting the RAGE pathway, highlighting their potential translational relevance in gynaecological practice.

## Introduction

The receptor for advanced glycation end-products (RAGE) is a complex multiligand receptor of the immunoglobulin superfamily that exists in living organisms in both transmembrane and soluble forms, each involved in distinct biological activities (Ref. 1). Its unique structure enables interaction with a wide variety of ligands, including advanced glycation end-products (AGEs) (Ref. 2), small α-helical proteins such as high mobility group box 1 protein (HMGB1) (Ref. 3), members of the S100 protein family (Ref. 4) and other molecules such as protein aggregates (e.g., β-amyloid), collagen types I and IV (Ref. 5), transmembrane proteins (e.g., Macrophage-1 antigen (MAC-1)) (Ref. 6), and components of the complement system (Ref. 7).

During early development, RAGE is highly expressed in various foetal tissues, including the skin (Ref. 8), muscles (Ref. 9), cartilage (Ref. 10), and the nervous system, where it may be involved in neurodifferentiation (Ref. 11). It is also strongly expressed in developing lung tissue, potentially contributing to alveolar type I cell proliferation and the process of alveolarization (Ref. 12). Interestingly, most adult tissues, except for the lungs (Ref. 13), express RAGE at low levels under normal physiological conditions (Ref. 14). However, in response to injury or pathological states such as chronic inflammation, both RAGE expression and the concentration of its ligands are significantly upregulated (Ref. 14).

Upon ligand binding, RAGE induces the phosphorylation of protein 38 mitogen-activated protein kinase (p38 MAPK), leading to enhanced activation of the nuclear factor kappa-light-chain-enhancer of activated B cells (NF-κB) signalling pathway. NF-κB activation upregulates the transcription of genes encoding inflammatory mediators such as tumour necrosis factor α (TNF-α) and interleukin-1beta (IL-1β), contributing to sustained tissue damage. Interestingly, NF-κB not only amplifies the expression of cytokines and tissue-damaging factors but also upregulates RAGE expression itself, thereby promoting further ligand–receptor interactions and sustaining the pro-inflammatory signalling cascade (Ref. 15).

Initially, RAGE was primarily associated with diabetes-related complications, particularly microangiopathy (Ref. 16). However, growing evidence indicates that RAGE also contributes to the pathogenesis of various conditions across multiple medical disciplines. In cardiology, for instance, it has been implicated in the development of coronary artery disease, the formation of atherosclerotic plaques (Ref. 17), myocardial fibrosis and the progression of heart failure (Ref. 18).

In oncology, RAGE and its ligands have been implicated in several critical processes, including tumour growth, promotion of metastasis, establishment of a tumour-supportive microenvironment and immune evasion. Their involvement has also been associated with an increased risk of metastasis and poor clinical prognosis for patients (Ref. 19).

In neurology, RAGE has been linked to the pathogenesis of various neurodegenerative disorders, such as Alzheimer’s disease, Parkinson’s disease and amyotrophic lateral sclerosis (ALS) (Ref. 20). Interestingly, RAGE may also be involved in neuromodulatory processes, such as the formation of the maternal–offspring bond, as it was shown in animal studies that mothers with low post-partum RAGE expression tend to neglect their offspring (Ref. 21). As a result, the first clinical trials with potent synthetic RAGE inhibitors, such as Azeliragon, have been initiated to evaluate their therapeutic potential in the treatment of Alzheimer’s disease (Ref. 22) and glioblastoma (Ref. 23).

Despite its recognized importance in several medical fields, the precise role of RAGE and its associated inflammatory pathways in gynaecological health and disease remains insufficiently characterized. Although the available data are limited, they strongly suggest that RAGE and its soluble isoforms may contribute to the development and progression of a range of gynaecological conditions. These include benign disorders of the endometrium and ovaries, pregnancy-related complications and gynaecological malignancies (Refs 24, 25, 26, 27). The potential involvement of RAGE in female reproductive health is perhaps not surprising, considering the dual role of inflammation in both physiological and pathological processes.

Inflammation is indispensable to key reproductive processes, including ovulation, menstruation, embryo implantation and labour onset. The precisely regulated release of inflammatory cytokines is essential for cyclic endometrial remodelling, while inflammatory interactions between the trophoblast and endometrium are critical for successful embryo implantation (Ref. 28). In contrast, dysregulated or chronic inflammation is associated with adverse pregnancy outcomes, including an increased risk of pregnancy complications and preterm birth (Ref. 29), and may be involved in the development of polycystic ovary syndrome (PCOS) (Ref. 30), endometriosis, and the progression of several gynaecological malignancies, including endometrial, cervical and ovarian cancers (Ref. 31).

Given the central role of inflammation in both physiological and pathological reproductive processes, as well as the well-established involvement of RAGE in inflammatory signalling, further research into its function may provide critical insights into the mechanisms underlying gynaecological disorders. In this comprehensive review, we examine the possible involvement of RAGE-mediated pathways in female reproductive health and disease and evaluate the potential of RAGE both as a diagnostic biomarker and as a target for emerging therapeutic strategies.

## Methodology of the review and literature selection process

Selected literature (*n* = 833) was retrieved from the National Center for Biotechnology Information database (https://www.ncbi.nlm.nih.gov/; accessed on 17th October 2024 and 10th February 2026). The search terms included: ‘RAGE’, ‘Cervix’, ‘Endometriosis’, ‘Endometrium’, ‘Fertility’, ‘Fibroid’, ‘Gynecology’, ‘Cancer’, ‘Disease’, ‘Inflammation’, ‘Infertility’, ‘Ovary’, ‘PCOS’, ‘Placenta’ and ‘Pregnancy’, combined in various configurations using the Boolean operators ‘AND’ and ‘OR’. Relevant literature was identified and analysed throughout manuscript preparation. The initial search was undertaken in November 2024, with update searches performed in April 2025 and February 2026 to identify newly published studies and ensure that relevant recent publications were not omitted. The final reference set includes literature available up to February 2026.

**Figure 1.**
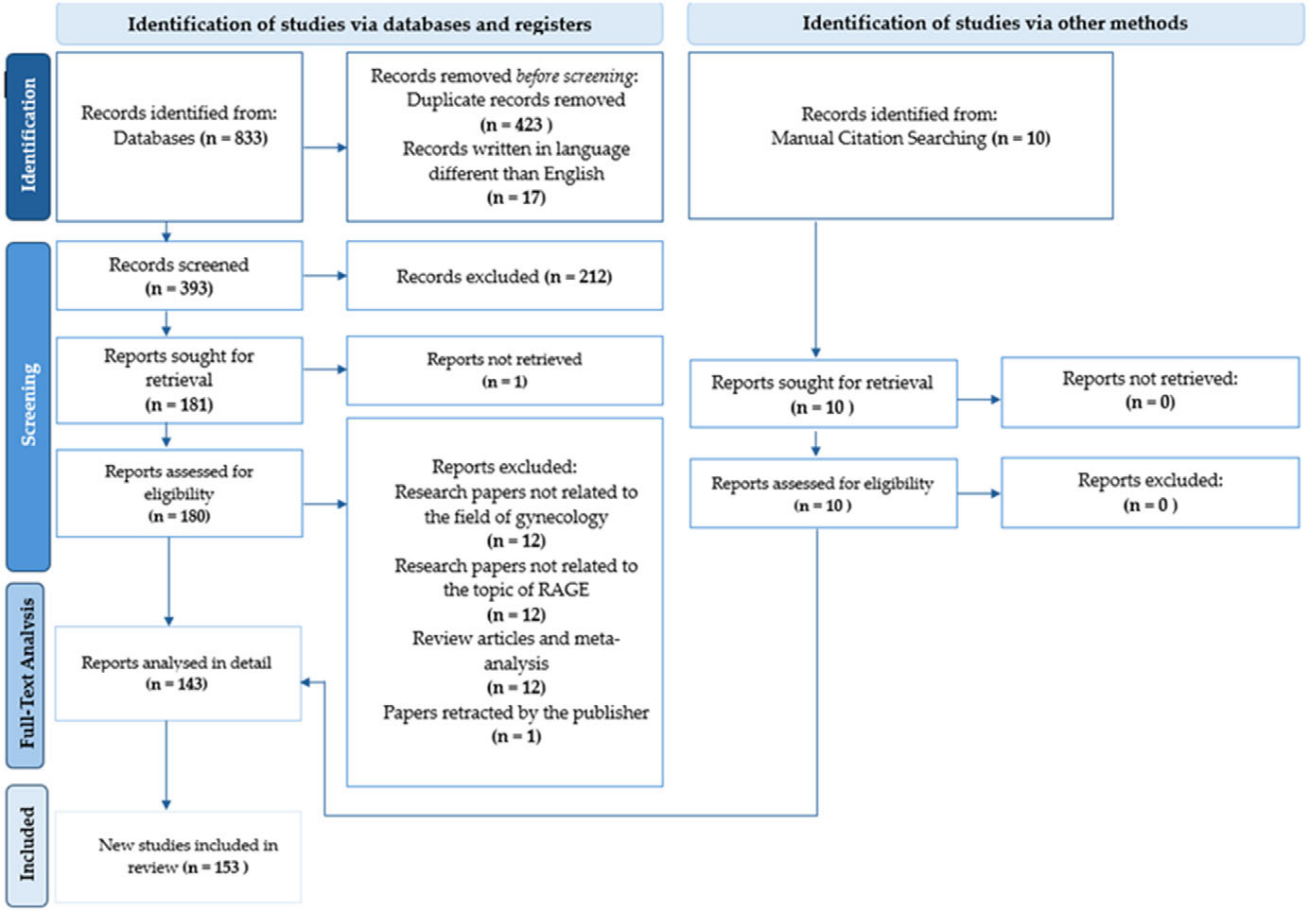
Flowchart illustrating the selection process of the analysed articles. Figure 1. long description.

A flowchart illustrating the literature selection process is presented in Figure 1. Initially, more than 800 papers were retrieved for screening. Articles published in the last 20 years were reviewed to provide a thorough overview of RAGE-centred research in gynaecology. In addition, three older studies were included after abstract review due to their relevance. The final study covered nearly three-quarters of publications published within the last decade. A preliminary selection was made based on titles and abstracts. Articles not meeting the inclusion criteria, specifically those not published in English or unrelated to RAGE in the context of gynaecology, were excluded. Additionally, 10 relevant publications were identified through manual searches and reference analysis of the included studies. As a result, 191 articles were deemed eligible for full-text review. Following a secondary screening, articles deemed irrelevant to the research topic were excluded, yielding a final selection of 153 studies, presented in Tables 1–12 of the manuscript.

**Table 1.** Overview of studies analysing RAGE expression in physiological states related to female reproductive health and pregnancy (↑ – increased concentration; ↓ – decreased concentration) Table 1. long description.

Year	Publication	State	Sample size	Control size	Material	Analysis method	Marker
2004	(Ref. 75)	Healthy pregnancy	N/A	N/A	Chorionic villi	IHC, Western blot	RAGE is present
2003	(Ref. 76)	Healthy pregnancy	10	10 (non-pregnant)	Myometrium, omental fat	IHC	↑RAGE
2020	(Ref. 77)	Healthy pregnancy	Foetal membrane zone of altered morphology (rupture zone)	Foetal membrane zone of intact morphology	Foetal membrane	IF, qRT-qPCR, Western blot	↑HMGB1, ↑RAGE (choriodecidua); ↑↑HMGB1, ↑↑RAGE (amnion)
2023	(Ref. 78)	Healthy pregnancy	N/A	N/A	Foetal membrane sample, primary amniotic epithelial cells	IF, qRT-qPCR, Western blot	RAGE is present
		Spontaneous labour at term	N/A	N/A			↑RAGE (amnion, primary amniotic epithelial cells)
2018	(Ref. 79)	Spontaneous labour at term	196 (spontaneous vaginal delivery)	49 (elective caesarean section), 42 (emergency caesarean section)	Cord venous blood	ELISA	↑HMGB1
			133 (spontaneous labour), 99 (induced labour)	55 (absent labour)			↑HMGB1
2014	(Ref. 74)	Endometrium in receptive phase	–	14 (healthy women)	endometrial biopsy, uterine fluid	IHC, iTRAQ mass spectrometry, Western blot	↓HMGB1, ↓S100A8,

Abbreviations: HMGB1: high mobility group box 1 protein; ELISA: enzyme-linked immunosorbent assay; IHC: immunohistochemistry; IF: immunofluorescence; N/A: not available; RAGE: receptor for advanced glycation end-products; qRT-PCR: quantitative real-time polymerase chain reaction.

**Table 2.** Summary of studies investigating the role of RAGE in endometrial pathologies (↑ indicates increased expression or concentration) Table 2. long description.

Year	Ref.	Disease	Sample size	Control size	Material	Analysis method	Marker
2023	(Ref. 86)	Endometriosis	24	0	Follicular fluid	ELISA	↑sRAGE (good embryo quality for IVF)
2017	(Ref. 57)	Endometriosis	84	55	Eutopic and ectopic endometrium, menstrual blood, peripheral blood (serum), peritoneal fluid	ELISA, IHC	No difference with control
2010	(Ref. 24)	Endometriosis	28	20	Endometriotic cyst, eutopic endometrium (isolated cells)	qRT-PCR, Western blot	↑RAGE, ↑ EN-RAGE
2010	(Ref. 88)	Endometriosis	12	10	Eutopic endometrium, ectopic endometrium (isolated cells)	qRT-PCR, Western blot	↑RAGE, ↑ EN-RAGE
2023	(Ref. 64)	Endometriosis	51	30	Peripheral blood (plasma), peritoneal fluid	Multiplex inflammation panel (Proseek)	↑EN-RAGE (peripheral blood plasma)
2019	(Ref. 58)	Endometriosis	30	20	Ectopic endometrium, peripheral blood (plasma)	ELISA, IHC	↑HMGB1(peripheral blood plasma, moderately fibrotic lesions), ↑RAGE (highly fibrotic lesions)
2008	(Ref. 85)	Endometriosis	8 (ectopic E.), 52 (plasma), 19 (P. fluid), 59 (F. fluid),	4 (eutopic E.), 72 (plasma), 35 (P. fluid), 78 (F. fluid)	Eutopic endometrium, ectopic endometrium, peripheral blood (plasma), peritoneal fluid, follicular fluid	ELISA, IHC, qRT-PCR,	↑sRAGE (follicular fluid)
2021	(Ref. 89)	Endometriosis (ovarian endometrioma)	10	0	Ovarian cyst wall	qRT-PCR, Western blot	↑HMGB1, ↑RAGE
2024	(Ref. 65)	Intrauterine adhesion – endometrial fibrosis	N/A	N/A	Eutopic endometrium	IHC, qRT-PCR, Western blot	↑S100A8, ↑S100A9, ↑RAGE
2022	(Ref. 87)	Intrauterine adhesion	6	16	Scar tissue, myometrium	RNA-Seq	↑AGE-RAGE

Abbreviations: AGE: advanced glycation end-product; ELISA: enzyme-linked immunosorbent assay; EN-RAGE: extracellular newly identified receptor for advanced glycation end-products binding protein; IHC: immunohistochemistry; HMGB1: high mobility group box 1 protein; N/A: not available; RAGE: receptor for advanced glycation end products, RNA-Seq: RNA sequencing, sRAGE: soluble receptor for advanced glycation end products, qRT-PCR: quantitative real-time polymerase chain reaction.

**Table 3.** Overview of clinical and experimental studies evaluating RAGE-related molecular changes in PCOS (↑ – increased concentration; ↓ – decreased concentration) Table 3. long description.

Year	Publication	Disease	Sample size	Control size	Material	Analysis method	Marker
2023	(Ref. 95)	PCOS	21	23	Granulosa lutein cells (from follicular fluid)	qRT-PCR, RNA-Seq	↑S100A9, ↑AGE-RAGE
2020	(Ref. 96)	PCOS	11	10	Primary granulosa-lutein cells and ovarian tissue	IHC, Western blot, qRT-PCR	↑RAGE, ↑AGEs
2007	(Ref. 25)	PCOS	6	6	Ovarian tissue biopsies	IHC	↑RAGE, ↑AGEs
2016	(Ref. 103)	PCOS	555	269	Peripheral blood	PCR-RFLP	RAGE polymorphisms –429T>C and –374T>A are not associated with PCOS
2005	(Ref. 104)	PCOS	29	22	Peripheral blood (serum, PBMCs)	ELISA, flow cytometry	↑RAGE, ↑AGEs
2008	(Ref. 105)	PCOS	100	25	Peripheral blood (serum)	ELISA	↑AGEs
2017	(Ref. 106)	PCOS	148	0	Peripheral blood (serum)	ELISA	↑AGEs, ↓sRAGE
2023	(Ref. 94)	PCOS	19	26	Peripheral blood (serum), follicular fluid	ELISA	No difference in sRAGE FF and blood serum levels
2024	(Ref. 107)	PCOS	4	4	Follicular fluid	Mass spectrometry	↓RAGE 344–355
2017	(Ref. 91)	PCOS	12	13	Follicular fluid	ELISA	↓sRAGE
2017	(Ref. 92)	PCOS	39	35	Follicular fluid	ELISA	↓sRAGE
2016	(Ref. 93)	PCOS	39	35	Follicular fluid	ELISA	↓sRAGE

Abbreviations: AGE: advanced glycation end-product; ELISA: enzyme-linked immunosorbent assay; EN-RAGE: extracellular newly identified receptor for advanced glycation end-products binding protein; IHC: immunohistochemistry; HMGB1: high mobility group box 1 protein; RAGE: receptor for advanced glycation end-products, RNA-Seq: RNA sequencing, sRAGE: soluble receptor for advanced glycation end-products; qRT-PCR: quantitative real-time polymerase chain reaction.

**Table 4.** Summary of studies investigating the role of RAGE in hypertensive disorders of pregnancy (↑ – increased concentration; ↑↑ – significantly increased concentration) Table 4. long description.

Year	Publication	Disease	Sample size	Control size	Material	Analysis method	Marker
2006	(Ref. 126)	Preeclampsia (PE)	10	10	Peripheral blood (serum), placental tissue	IHC, qRT-PCR, Western blot	↑RAGE, ↑AGEs
2007	(Ref. 127)	Preeclampsia (PE)	13	12	Placental tissue	IHC, In situ hybridization	↑HMGB1
2011	(Ref. 119)	Hypertensive disorders	60	32	Placental tissue, peripheral blood (plasma)	ELISA, IHC	↑RAGE, ↑S100A8/S100A9
2011	(Ref. 120)	Preeclampsia (PE)	13 (pregnant women with hypertension), 38 (pregnant women with preeclampsia)	16 (non-pregnant controls), 68 (healthy pregnant controls)	Peripheral blood, amniotic fluid, cord blood, placental and amniochorion tissue	ELISA, qRT-PCR	↑sRAGE, ↑esRAGE (maternal blood serum and amniotic fluid), ↑RAGE (amniochorion)
2015	(Ref. 26)	Preeclampsia (PE)	64	61	Placental tissue	ELISA, IF, IHC, Western blot	↑HMGB1, ↑RAGE
2016	(Ref. 115)	Preeclampsia (PE)	25	110	Placental tissue	IHC, Western blot	↑HMGB1
		Severe preeclampsia (PE)	49				↑↑HMGB1
2016	(Ref. 116)	Preeclampsia (PE)	N/A	N/A	Placental tissue	IHC, qRT-PCR, Western blot	↑RAGE (protein level)
2016	(Ref. 128)	Preeclampsia (PE)	21	21	Trophoblast, peripheral blood	ELISA, IHC, Western blot	↑HMGB1, RAGE not affected
2017	(Ref. 112)	Preeclampsia (PE)	18	19	Placental tissue	IF, immunoprecipitation, qRT-PCR, Western blot	↑HMGB1
2017	(Ref. 121)	Preeclampsia (PE)	32	30	Placental Tissue, Maternal Peripheral Blood, Umbilical blood	ELISA, IHC, qRT-qPCR, Western Blot	↑RAGE (placental tissue), ↑AGEs (placental tissue, umbilical blood, maternal blood), ↑sRAGE (maternal blood), ↓sRAGE(umbilical blood)
2021	(Ref. 113)	Preeclampsia (PE)	6	6	Placental tissue	IF, Western blot	↑RAGE
2023	(Ref. 48)	Preeclampsia (PE)	3	3	Placental tissue	ELISA, Western blot	No differences were found
2003	(Ref. 76)	Preeclampsia (PE)	11	9 (non-pregnant)	Myometrium, omental fat	IHC	↑↑RAGE
2010	(Ref. 123)	Preeclampsia (PE)	18	79	Peripheral blood	ELISA	↑sRAGE
2011	(Ref. 124)	Preeclampsia (PE)	28	87	Peripheral blood	ELISA	↑esRAGE, ↑esRAGE/sRAGE ratio
2012	(Ref. 129)	Preeclampsia (PE)	16	16	Peripheral blood	ELISA	↑HMGB1
2012	(Ref. 118)	Preeclampsia (PE)	17	33	Peripheral blood	ELISA	↑HMGB1, ↑S100A12 (preeclampsia); ↓S100A12 (2nd, 3rd trimester healthy pregnancy)
2012	(Ref. 122)	Preeclampsia (PE)	35	120	Peripheral blood	ELISA, genotyping	No differences in RAGE Polymorphism, ↑sRAGE
2014	(Ref. 114)	Preeclampsia (PE)	16	16 (3rd trimester pregnant without PE), 16 (non-pregnant)	Peripheral blood	ELISA	↑HMGB1 (3rd trimester pregnancy), ↑↑ (preeclampsia)
2024	(Ref. 117)	Preeclampsia (PE)	56	56	Peripheral blood	ELISA	↑HMGB1, ↑RAGE
		Preeclampsia (PE) with acute heart failure	40				↑↑HMGB1, ↑↑RAGE

Abbreviations: AGEs: advanced glycation end-products; ELISA: enzyme-linked immunosorbent assay; esRAGE: endogenous secretory receptor for advanced glycation end-products; IF: immunofluorescence; IHC: immunohistochemistry; HMGB1: high mobility group box 1 protein; PE: preeclampsia; RAGE: receptor for advanced glycation end-products; sRAGE: soluble receptor for advanced glycation end-products; qRT-PCR: quantitative real-time polymerase chain reaction

**Table 5.** Summary of studies investigating the role of RAGE in pregnancy-associated metabolic conditions (*↑ –* increased concentration*; ↓ –* decreased concentration) Table 5. long description.

Year	Publication	Disease	Sample size	Control size	Material	Analysis method	Marker
2021	(Ref. 113)	Gestational diabetes mellitus	6	6	Placental tissue	IF, Western blot	RAGE not affected
2010	(Ref. 140)	Gestational diabetes mellitus	150	600	Peripheral blood	PCR-RFLP	RAGE polymorphisms –429T>C and –374T>A are not associated with gestational diabetes
2019	(Ref. 134)	Gestational diabetes mellitus	12	12	Foetal membrane, maternal omental adipose tissue, maternal peripheral blood	ELISA, Western blot	↑HMGB1 (foetal membranes), ↑RAGE (omental tissue)
2019	(Ref. 136)	Gestational diabetes mellitus, vascular inflammation	N/A	N/A	HuVECs from umbilical vein, peripheral blood (plasma)	Western blot	↑RAGE, ↑AGEs
2023	(Ref. 48)	Gestational diabetes mellitus	3	3	Placental tissue	ELISA, Western blot	RAGE not affected
2020	(Ref. 137)	Gestational diabetes mellitus	N/A	N/A	Umbilical cord	IF, Western blot	↑RAGE, ↑AGEs
2016	(Ref. 116)	Gestational diabetes mellitus	N/A	N/A	Placental tissue	IHC, qRT-qPCR, Western blot	↓RAGE (mRNA level), ↓RAGE (protein level)
2021	(Ref. 133)	Impaired glucose tolerance	50	71	Peripheral blood	ELISA	↑AGEs, ↑RAGE, ↑CML
		Gestational diabetes mellitus	59	71	Peripheral blood	ELISA	↑↑AGEs, ↑↑RAGE, ↑↑CML
2009	(Ref. 138)	Type I diabetes mellitus during pregnancy	29	29	Peripheral blood	ELISA	↓sRAGE, ↑AGEs
2024	(Ref. 141)	Hyperglycaemia	N/A	N/A	Human ovarian granulosa (GCN–01) cell line	qRT-PCR	↑RAGE
2018	(Ref. 135)	Obesity	16 (Obese women)	17 (Lean women)	Uterine fluid, endometrial biopsy	IHC, ELISA	↑RAGE, ↑AGEs, ↑CML [Ref. AGEs]

Abbreviations: AGEs: advanced glycation end-products; CML: carboxymethyllysine; ELISA: enzyme-linked immunosorbent assay; IHC: immunohistochemistry; PCR-RFLP: polymerase chain reaction-restriction fragment length polymorphism; RAGE: receptor for advanced glycation end-products; sRAGE: soluble receptor for advanced glycation end-products; qRT-PCR: quantitative real-time polymerase chain reaction.

**Table 6.** Summary of studies evaluating RAGE-related molecular markers in pathologies of foetal membranes and preterm birth (↑ – increased concentration; ↓ – decreased concentration) Table 6. long description.

Year	Publication	Disease	Sample size	Control size	Material	Analysis method	Marker
2018	(Ref. 145)	Preterm premature rupture of the membranes (pPROM)	60	60	Placental biopsy, peripheral blood	ELISA, IF, qRT-PCR, Western blot	↑HMGB1, ↑RAGE
		Premature rupture of the membranes (PROM)	60	60	Placental biopsy, peripheral blood	ELISA, IF, qRT-PCR, Western blot	No difference
2015	(Ref. 146)	Threatened premature labour	41	N/A	Peripheral blood	ELISA	No difference
		Preterm premature rupture of the membranes (pPROM)	49	N/A			↑esRAGE
		Preterm rupture of membranes at term	48	N/A			↑esRAGE
2021	(Ref. 147)	Rupture of membranes during threatened preterm labour	38	145	Amniotic fluid	ELISA, antibody-based protein microarray	↑EN-RAGE
2020	(Ref. 160)	Preterm premature rupture of the membranes (pPROM)	82	164	Peripheral blood (plasma), serum-extracted exosomes	ELISA	No difference in AGEs, sRAGE, HMGB1, S100A8/A9 concentrations
2009	(Ref. 161)	Chorioamnionitis and preterm premature rupture of the membranes (pPROM)	38	24	Amniotic fluid, cord blood (serum)	ELISA, mass spectrometry,	No difference
		Severe chorioamnionitis and preterm premature rupture of the membranes (pPROM)	59				↓sRAGE
2008	(Ref. 152)	Chorioamnionitis	82	122	Amniotic fluid	ELISA	↑esRAGE, ↑sRAGE
2012	(Ref. 162)	Chorioamnionitis	46	115	Amniotic fluid	ELISA	↑HMGB1, ↓sRAGE
2022	(Ref. 150)	Histologic chorioamnionitis in pPROM	15 (marker selection), 100 (marker validation)	15 (marker selection)	Amniotic fluid	ELISA, antibody-based protein microarray	↑EN-RAGE, ↑S100 A8/A9
2016	(Ref. 149)	Chorioamnionitis and preterm labour	100	165	Amniotic fluid, placental tissue sample, foetal membrane sample	ELISA, IHC, mass spectrometry	↑S100A12/EN-RAGE, ↑S100A8, ↑HMGB1,
2007	(Ref. 151)	Positive culture chorioamnionitis	27	59	Amniotic fluid, placental tissue sample, foetal membrane sample	ELISA, IHC, qRT-PCR, SELDI-TOF	↑↑S100A12/EN-RAGE, ↓sRAGE
		Negative culture chorioamnionitis	27				↑S100A12/EN-RAGE, ↓sRAGE
2016	(Ref. 154)	Spontaneous preterm labour	19	21	Chorioamniotic membrane samples	ELISA	↑HMGB1
2023	(Ref. 153)	Spontaneous preterm labour	65	0	Amniotic fluid	ELISA	↑EN-RAGE
2022	(Ref. 163)	Spontaneous preterm labour	20 (marker selection), 267 (marker validation)	20	Amniotic fluid	ELISA, antibody-based protein microarray	↑EN-RAGE, ↑S100 A8/A9
2010	(Ref. 123)	Spontaneous preterm labour	24	79	Peripheral blood	ELISA	↓sRAGE
2010	(Ref. 164)	Spontaneous preterm labour	20	24	Cervical biopsy	IHC, ELISA, qRT-PCR,	No difference in RAGE, sRAGE, HMGB1 levels
2016	(Ref. 157)	Threatened premature labour	138	73	Peripheral blood	ELISA	↓sRAGE
2008	(Ref. 156)	Threatened preterm labour and premature rupture of the membranes (PROM)	46	35 (healthy pregnancy), 15 (non-pregnancy)	Peripheral blood (serum)	ELISA	↑sRAGE (in threatened preterm labour) ↓sRAGE (in women with pPROM)
2012	(Ref. 122)	Threatened preterm labour	99	120	Peripheral blood	ELISA, polymorphism analysis	No difference in RAGE polymorphism
2021	(Ref. 113)	Preterm labour	6	6	Formalin-fixed, parafin-embedded placental tissue	IF, Western blot	↑RAGE
2023	(Ref. 78)	Pregnancy	N/A	N/A	Foetal membrane sample	IF, qRT-PCR, Western blot	No difference
		Term in spontaneous labour	N/A	N/A			No difference
		Term without labour	N/A	N/A			↑RAGE
2012	(Ref. 155)	Preterm labour	498	753	Maternal peripheral blood (serum), cord blood (serum)	ELISA	↓sRAGE
2021	(Ref. 158)	Inflammation induced by maternal smoking	N/A	N/A	Explants from amnion and choriodecidua	Western blot	↑HMGB1 (amnion)
2022	(Ref. 159)	Placental infection with Zika virus	11	11	Placental sample	IF, quantitative proteomics, Western blot	↑RAGE (placental tissue), ↓RAGE (trophoblast cells)

Abbreviations: ELISA: enzyme-linked immunosorbent assay; EN-RAGE: extracellular newly identified receptor for advanced glycation end-products binding protein; esRAGE: endogenous secretory receptor for advanced glycation end-products; IF: immunofluorescence; IHC: immunohistochemistry; HMGB1: high mobility group box 1 protein; PROM: premature rupture of membranes; pPROM: preterm premature rupture of membranes; RAGE: receptor for advanced glycation end-products, SELDI-TOF: surface-enhanced laser desorption/ionization time-of-flight mass spectrometry; sRAGE: soluble receptor for advanced glycation end-products; qRT-PCR: quantitative real-time polymerase chain reaction.

**Table 7.** Studies analysing the role of RAGE in pregnancy loss (↑ – increased concentration; ↓ – decreased concentration) Table 7. long description.

Year	Publication	Disease	Sample size	Control size	Material	Analysis method	Marker
2022	(Ref. 166)	Recurrent spontaneous abortion	31	26	Trophoblast tissue	Flow cytometry, qRT-PCR, Western blot,	↓RAGE
2017	(Ref. 167)	Recurrent spontaneous abortion	60	20	Peripheral blood	ELISA, Protein antibody microarray	↓RAGE
2014	(Ref. 170)	Recurrent spontaneous abortion	63	30	Peripheral blood	ELISA	↑sRAGE
2020	(Ref. 168)	Unexplained recurrent spontaneous abortion	55	55	Villus and decidual tissue, peripheral blood	IF, IHC, ELISA, Western blot	↑HMGB1, ↑RAGE
2021	(Ref. 169)	Unexplained Recurrent Spontaneous Abortion	75	75	Decidual tissue	Western blot	↑HMGB1

Abbreviations: ELISA: enzyme-linked immunosorbent assay; IF: immunofluorescence; IHC: immunohistochemistry; HMGB1: high mobility group box 1 protein; RAGE: receptor for advanced glycation end-products; sRAGE: soluble receptor for advanced glycation end-products; qRT-PCR: quantitative real-time polymerase chain reaction

**Table 8.** Studies analysing the role of RAGE in the pathophysiology of infant morbidities (↑ – increased concentration; ↓ – decreased concentration) Table 8. long description.

Year	Publication	Disease	Sample size	Control size	Material	Analysis method	Marker
2023	(Ref. 48)	Intrauterine growth restriction (IUGR)	3	3	Placenta	ELISA, Western blot	RAGE not effected
2012	(Ref. 122)	Intrauterine growth restriction (IUGR)	22	120	Peripheral blood	ELISA, polymorphism analysis	No differences
2016	(Ref. 116)	Intrauterine growth restriction (IUGR)	N/A	N/A	Placenta	IHC, qRT-qPCR, Western blot	↓RAGE (protein)
2013	(Ref. 172)	Infant morbidities	130	N/A	Infant peripheral blood	ELISA	↑sRAGE, ↑S100B (maternal chorioamnionitis), ↓sRAGE (sepsis, respiratory failure, maternal preeclampsia)
2017	(Ref. 173)	Brain injury in preterm infants	44	67	Foetal membranes, umbilical cord blood	ELISA, IHC	↑HMGB1, ↑RAGE, ↓sRAGE,
2012	(Ref. 122)	Intrahepatic pregnancy cholestasis	14	120	Peripheral blood	ELISA, polymorphism analysis	No differences
2020	(Ref. 174)	Intrauterine HBV infection	16	26	Neonate peripheral blood, placenta	IHC, mass spectroscopy proteomics, Western blot	↑S100A8, ↑S100A9, ↑S100A12

Abbreviations: ELISA: enzyme-linked immunosorbent assay; IHC: immunohistochemistry; IUGR: intrauterine growth restriction; HMGB1: high mobility group box 1 protein; RAGE: receptor for advanced glycation end-products; sRAGE: soluble receptor for advanced glycation end-products; qRT-PCR: quantitative real-time polymerase chain reaction

**Table 9.** Studies examining the presence and regulation of RAGE in gynaecology-related states (↑ – increased concentration; ↓ – decreased concentration) Table 9. long description.

Year	Publication	Disease	Sample size	Control size	Material	Analysis method	Marker
2019	(Ref. 175)	Antiphospholipid syndrome (APS)	60	30	Peripheral blood (serum)	Western blot	↑HMGB1, ↑sRAGE
2017	(Ref. 176)	Antiphospholipid syndrome (APS)	30	30	Peripheral blood (serum)	Western blot	↑HMGB1, ↑sRAGE
2018	(Ref. 177)	Pelvic organ prolapse	20	10	Vaginal tissue	IHC, Western blot	↑AGEs,
2017	(Ref. 178)	Pelvic organ prolapse	3	3	Vaginal tissue	qRT-PCR, Western blot	↑RAGE
2015	(Ref. 179)	Pelvic organ prolapse	44	46	Vaginal tissue	IHC, genotyping, Western blot	↑AGEs
2022	(Ref. 180)	Acute cervical insufficiency	50	0	Amniotic fluid	ELISA	↑RAGE
2022	(Ref. 181)	Cervical insufficiency	80	49	Amniotic fluid	Antibody-based protein microarray, ELISA	↑EN-RAGE, ↑S100 A8/A9
2014	(Ref. 185)	Ovarian ageing	N/A	N/A	Primary ovarian granulosa–lutein cells, primary ovarian monocytes	IF, flow cytometry	↑AGEs, ↑RAGE
2014	(Ref. 183)	Diminished ovarian reserve	33	31	Follicular fluid, peripheral blood (serum)	ELISA	↓sRAGE (follicular fluid)
2017	(Ref. 184)	Infertility, advanced maternal age (AMA)	62 (young infertile women), 62 (advanced maternal age women)	0	Follicular fluid	ELISA	↓sRAGE
2013	(Ref. 186)	IVF embryo quality	26 (poor-quality embryo)	19 (High Quality Embryo)	Follicular fluid	Luminex xMAP	↓sRAGE (high-quality embryo)
2010	(Ref. 182)	Ageing	114 (elder women, age > 35 years)	77 (Young Women, Age < 35 Years)	Peripheral blood (plasma), follicular fluid	ELISA	↓sRAGE (blood plasma)

Abbreviations: AGEs: advanced glycation end-products, AMA: advanced maternal age, APS: antiphospholipid syndrome, HMGB1: high mobility group box 1 protein, ELISA: enzyme-linked immunosorbent assay, EN-RAGE: extracellular newly identified receptor for advanced glycation end-products binding protein, IF: immunofluorescence, IHC: immunohistochemistry, IVF: in vitro fertilization, RAGE: receptor for advanced glycation end-products, sRAGE: soluble receptor for advanced glycation end-products, qRT-PCR: quantitative real-time polymerase chain reaction

**Table 10.** Studies examining RAGE expression in gynaecological malignancies (↑ – increased concentration; ↑↑ – significantly increased; ↑↑↑ – substantially increased; ↓ – decreased concentration) Table 10. long description.

Year	Publication	Disease	Sample size	Control size	Material	Analysis method	Marker
2013	(Ref. 194)	Chronic cervicitis	30	0	Formalin-fixed, parafin-embedded cervical tissue sample	IHC	↑S100A9, ↑RAGE
		Cervical intraepithelial neoplasia	50	0	Formalin-fixed, parafin-embedded cervical tissue sample	IHC	↑↑S100A9, ↑↑RAGE
		Squamous cervical cancer	40	0	Formalin-fixed, parafin-embedded cervical tissue sample	IHC	↑↑↑S100A9, ↑↑↑RAGE
2021	(Ref. 205)	Chronic cervicitis	Not related – HCE cell line	Not related – HCE cell line	HCE cell line	Western blot	↑HMGB1, ↑RAGE
2017	(Ref. 193)	Cervical intraepithelial neoplasia	88	24	Formalin-fixed, paraffin embedded tissue microarray	IHC	↑S100A7
		Cervical cancer	51	24	Formalin-fixed, paraffin embedded tissue microarray	IHC	↑S100A7
2017	(Ref. 192)	Cervical cancer	99	24	Tissue samples from surgery	IHC	↑HMGB1
2021	(Ref. 191)	Cervical cancer	N/A	N/A	Tumour tissue	Western blot, IHC, IF	↑RAGE
2018	(Ref. 206)	Cervical cancer	201	320	Peripheral blood	SNP genotyping, RT-PCR	↓GT/TT genotype of rs184003
2012	(Ref. 195)	Cervical cancer	448	715	Peripheral blood	SNP genotyping, ELISA	↓sRAGE, 82G>S polymorphism of RAGE gene may be associated with the susceptibility of cervical cancer
2013	(Ref. 196)	Epithelial ovarian cancer	190	210	Peripheral blood	RAGE genotyping – PCR-RFLP	82G>S polymorphism of RAGE gene may be associated with the susceptibility of EOC
2017	(Ref. 198)	Ovarian cancer	Not related – A2780 cell line	Not related – A2780 cell line	A2780 cell line	qPCR, Western blot, IF, flow cytometry	↑S100B, ↑RAGE
			338	24	Data from GEO database	Bioinformatic analysis	↑S100B
2023	(Ref. 27)	Ovarian cancer	76	68	Peripheral blood (serum)	ELISA	↑↑HMGB1, ↓↓sRAGE
2015	(Ref. 197)	Serous ovarian carcinoma	9	15	Tissue samples from surgery	IHC	↑RAGE
2017	(Ref. 198)	Ovarian cancer	338	24	Data from GEO database	Bioinformatic analysis	↑S100B
2016	(Ref. 200)	Endometrial cancer	72	20	Endometrial tissue samples from surgery	IHC	↑RAGE
2021	(Ref. 201)	Endometrial adenocarcinoma	10	12	Endometrial tissue from endometrial curettage	Western blot, IF, IHC	↑RAGE
2017	(Ref. 203)	Endometrial adenocarcinoma	240	60	Endometrial tissue samples from surgery, primary and established endometrial and endometrial cancer cell lines	Western blot, IHC, qPCR	↓HMGB1
2019	(Ref. 202)	Endometrial cancer	91	70	Endometrial biopsy, endometrial cancer cell line	IHC, RT-qPCR, IF,	↑RAGE

Abbreviations: HMGB1: high mobility group box 1 protein, ELISA: enzyme-linked immunosorbent assay, IF: immunofluorescence, IHC: immunohistochemistry, PCR–RFLP: polymerase chain reaction–restriction fragment length polymorphism, RAGE: receptor for advanced glycation end-products, qRT–PCR: quantitative real-time polymerase chain reaction, SNP genotyping: single-nucleotide polymorphism genotyping, sRAGE: soluble receptor for advanced glycation end-products, qPCR: quantitative real-time polymerase chain reaction.

**Table 11.** Studies analysing RAGE expression in animal models of gynaecological and pregnancy-related disorders (↑ indicates increased concentration; ↓ indicates decreased concentration) Table 11. long description.

Year	Publication	Species	Disease	Sample size	Control size	Material	Analysis method	Marker
2020	(Ref. 207)	Rats	Gestational diabetes mellitus	20	10	Peripheral blood, placenta	ELISA, qRT-PCR	↑AGEs, ↑RAGE,
2024	(Ref. 208)	Rats	Gestational diabetes mellitus	24	6	Peripheral blood, placenta, foetal tissues	ELISA, qRT-PCR	↑RAGE, ↑AGEs
2014	(Ref. 209)	Rabbit	Gestational diabetes mellitus	N/A	N/A	Maternal blood, maternal uterine tissue, blastocysts	IHC, HPLC/mass spectrometry, qRT-qPCR, slot blot	↑AGEs, ↑RAGE
2021	(Ref. 210)	Rats	Gestational diabetes mellitus	N/A	N/A	Placenta, Heart tissue, brain tissue	qRT-PCR	↑RAGE
2015	(Ref. 211)	Rats	Gestational diabetes mellitus	10	10	Neonatal brain tissue	Western blot	↑RAGE
2020	(Ref. 212)	Mice	PCOS	N/A	N/A	Peripheral blood	ELISA	↑RAGE
2023	(Ref. 213)	Mice	PCOS	N/A	N/A	Mouse uterus	Western blot	↑MG-AGEs, ↓RAGE
2020	(Ref. 214)	Mice	PCOS	10	10	Ovarian tissue	Western blot	↑RAGE
2021	(Ref. 215)	Pig	Intra-amniotic infection	3	3	Amniotic fluid and amniotic membrane	ELISA, IF, qRT-PCR	↓RAGE, ↓HMGB1 (amniotic membrane); ↑HMGB1, ↓sRAGE (amniotic fluid)
2024	(Ref. 216)	Mare	Endometritis	N/A	N/A	Uterine fluid	Fluorescent beads based multiplex assay, qRT-PCR	No difference
2020	(Ref. 217)	Mice	Prenatal cleft palate	44	36	Maternal peripheral blood, mouse placental, mouse embryos	ELISA, IHC	↑sRAGE
2017	(Ref. 218)	Mice	Prenatal cleft palate	60	30	Amniotic fluid, mouse embryos	ELISA, IHC, protein antibody-based microarray	↓sRAGE
2021	(Ref. 219)	Mice	Prenatal lung development after nicotine exposure	24	7	Mouse offspring lungs	IHC, Western blot	↑HMGB1, ↑RAGE
2015	(Ref. 220)	Rats	Foetal development in gestational diabetes mellitus	30	6	Placental and foetal tissues	IHC, ELISA, qRT-PCR, Western blot	↑AGEs, ↑RAGE
2019	(Ref. 221)	Bovine	Cellular ageing	7	5	Primary oviduct epithelial cells	qRT-PCR	↑S100A8/S100A9
2024	(Ref. 222)	Mice	Decidualization and implantation	28	0	Uterine tissue sample, mouse blastocysts	IHC, in situ hybridization, ELISA, qRT-PCR, Western blot	↑RAGE, ↑S100A6
2017	(Ref. 223)	Mice	Intrauterine growth restriction induced by second-hand smoking	N/A	N/A	Mouse placental tissues	Western blot	↑RAGE
2016	(Ref. 224)	Mice	Diabetic embryopathy	N/A	N/A	Peripheral blood (plasma), mouse foetus	ELISA, qRT-PCR	↑RAGE, ↑CML(AGEs)

Abbreviations: AGEs: advanced glycation end-products, CML: carboxymethyllysine, ELISA: enzyme-linked immunosorbent assay, IF: immunofluorescence,HMGB1: high mobility group box 1 protein, IHC: immunohistochemistry, MG: methylglyoxal, N/A: not available, RAGE: receptor for advanced glycation end-products, sRAGE: soluble receptor for advanced glycation end-products, qPCR: quantitative real-time polymerase chain reaction.

**Table 12.** Therapeutic approaches targeting RAGE in gynaecological diseases (↑ indicates increased concentration; ↓ indicates decreased concentration) Table 12. long description.

Year	Publication	Condition	Model	Medication	Type of medication	Outcome
2020	(Ref. 259)	PCOS	Bioinformatic analysis	Cangfu Daotan Decoction	Herbal medicine – non-specific RAGE modulator	↓AGE-RAGE pathway
2023	(Ref. 260)	PCOS	Bioinformatic analysis	Leonuri Herba	Herbal medicine – non-specific RAGE modulator	↓AGE-RAGE pathway
2018	(Ref. 261)	PCOS	Bioinformatic analysis	Erxian Decoction	Herbal medicine – non-specific RAGE modulator	↓AGE-RAGE pathway, could alleviate the symptoms of PCOS
2023	(Ref. 262)	PCOS	Bioinformatic analysis	Angelica Sinensis – Radix Rehmanniae	Herbal medicine – non-specific RAGE modulator	↓AGE-RAGE pathway, improves hyperandrogen status, regulates glucose homeostasis, corrects lipid metabolism
2020	(Ref. 212)	PCOS	PCOS mouse model, human ovarian granulosa cell line	Pigment epithelium-derived factor (PEDF)	Recombinant protein	↓Weight gain, ↓IL6, ↓IL8, ↓RAGE, ↓AMH, ↓VEGF
2023	(Ref. 213)	PCOS	PCOS mouse model	L-carnitine	Non-specific RAGE modulator	↓Oxidative stress, ↓Mitochondrial damage, ↓RAGE, ↓AGEs
2020	(Ref. 214)	PCOS	PCOS mouse model	L-carnitine	Non-specific RAGE modulator	↓AGEs, restore of the normal estrous cyclicity, ↓Testosterone, ↑Number of ovulated oocytes, ↑Oocyte quality, ↓Oocyte fibrosis, ↓AGEs, ↓RAGE
2023	(Ref. 241)	Premature ovarian failure	Bioinformatic analysis, cyclophosphamide-induced primary ovarian insufficiency mouse model	Zishen Yutai Pill	Herbal medicine – non-specific RAGE modulator	↓Apoptosis, ↑AMH, ↓Atretic follicles number, ↑Primary and secondary follicles number, ↓AGE-RAGE
2023	(Ref. 242)	Premature ovarian failure	Cisplatin-induced premature ovarian failure rats, senescent human ovarian granulosa cell	Erxian decoction	Herbal medicine – non-specific RAGE modulator	↓LH, ↓FSH, ↑E2, ↑AMH, ovarian index, follicle count, proportion of atretic follicles, ↓ RAGE, ↑Antioxidant capabilities, ↑Anti-apoptotic capabilities
2024	(Ref. 250)	Premature ovarian failure	Mouse model of galactose-induced premature ovarian insufficiency	Metformin	Drug	↓PI3K, ↓Akt, ↓FOXO3a, ↓RAGE, ↓AGEs, ↑Primordial follicles ratio, ↓Atretic follicles number, ↓Apoptotic-related cleaved caspase–3 expression
2023	(Ref. 244)	Ovarian dysfunction	Primary ovarian granulosa cells	Pyridoxamine	Vitamin – non-specific RAGE modulator	↓RAGE, ↑Oestrogen (E2), ↓Progesterone, ↓Testosterone, reversed changes caused by human glycated albumin (HGA)
2024	(Ref. 246)	Diminished ovarian reserve	Bioinformatic analysis, human ovarian granulosa cell line	Indole–3-propionic acid	Anti-oxidative agent, non-specific RAGE modulator	↓Oxidative stress, ↓Reduced cellular damage, ↓TNF expression
2024	(Ref. 243)	Ovarian granulosa cells hormonal dysfunction	Primary ovarian granulosa cells treated with human glycated albumin	Alpha-lipoic acid	Anti-oxidative agent, non-specific RAGE modulator	↓P4, ↑E2, ↓RAGE, ↓STAR, ↓3β-HSD, ↓17β-HSD, ↑CYP19A1, corrected steroid hormones profile and enzyme expression levels
2018	(Ref. 245)	Ovarian granulosa cells hormonal dysfunction	Human granulosa cells	1,25-dihydroxyvitamin D3	Vitamin – non-specific RAGE modulator	↓RAGE, ↓CYP11A1, ↓StAR, ↓CYP17A1, ↓LHR
2019	(Ref. 263)	AGEs influence on anti-mullerian hormone expression	Human granulosa cells	1,25-dihydroxyvitamin D3	Vitamin – non-specific RAGE modulator	↓AMHR–2, ↓SMAD 1/5/8 phosphorylation, reversed changes caused by AGEs
2024	(Ref. 141)	Hyperglycaemia included ageing	Testicular and ovarian cell lines	Betaine	Anti-oxidative agent, non-specific RAGE modulator	↓AGEs, ↓Cellular ageing and senescence related signalling genes
2023	(Ref. 247)	Endometriosis	Bioinformatic analysis, endometrial transplantation mouse model, 12z endometriosis cell line	Neferine	Naturally occurring alkaloid non-specific RAGE modulator	↓Fibronectin, ↓Collagen I, ↓Connective tissue growth factor, ↓Smooth muscle actin, ↓Endometriosis – related fibrosis, ↓12z Invasive potential, ↓Endometriosis progression
2021	(Ref. 248)	Endometriosis	Bioinformatic analysis, hEM15a endometrial stromal cell line	Guizhi Fuling Wan	Herbal medicine – non-specific RAGE modulator	↓Proliferation rate, ↑Apoptosis, ↑P53, ↑BAX, ↑Caspase3
2020	(Ref. 249)	Pelvic inflammatory disease (PID)	Bioinformatic analysis, macrophage RAW264.7 cell line	Sargentodoxa cuneata, Patrinia scabiosifolia	Herbal medicine – non-specific RAGE modulator	↓Macrophage proliferation, ↓Release of NO by macrophages, ↓TNF-α release
2017	(Ref. 178)	Pelvic organ prolapse	Primary human vaginal fibroblasts	siRNA RAGE silencing	Genetic modifications	↓p38 MAPK, ↓NF-κB-p-p65, ↑Collagen I, ↓MMP I, ↓RAGE
2021	(Ref. 205)	Cervical inflammation	Cervical epithelial cell line	FPS-ZM1	RAGE inhibitor	↓Proliferation rate, ↓Cyclin D1, ↓Ki67, ↓Cellular migration rate, ↓Apoptosis rate, ↓Inflammation grade, ↓Pyroptosis, ↓IL–1β,↓ IL–6, ↓TNF-α, ↓TLR4, ↓NF-κB p-p65
2020	(Ref. 207)	Gestational diabetes mellitus	Streptozotocin-induced diabetes mellitus rats	Low molecular weight heparin (nadroparin)	Drug	↓RAGE, ↓NF-κB, ↓VEGF, ↓Placental permeability, ↑ZO–1, ↑OCLN
2021	(Ref. 251)	Gestational diabetes mellitus – foetal development	Streptozotocin-induced diabetes mellitus rats	Crocetin	Naturally occurring polyphenol non-specific RAGE modulator	↑Body weight, ↑Foetus weight, ↓Blood glucose level, ↓AGEs, ↓Triglycerides, ↓Total cholesterol, ↓LDL, ↓VLDL, ↑HDL, ↓CRP, ↓HbA1c, ↓IL–6, ↓TNF-α, ↓IL–1β,↓RAGE, ↓p65
2014	(Ref. 220)	Gestational diabetes mellitus – foetal development	Streptozotocin-induced diabetes mellitus rats	sRAGE	Recombinant protein	↓Foetal developmental defect, ↓AGEs, partially restored cytoplasmic localization of p65, ↓RAGE, ↓NOX2, ↓MCP–1, EGFR, ↓VCAM–1 ↓p65
2024	(Ref. 208)	Gestational diabetes mellitus – foetal development	Streptozotocin-induced diabetes mellitus rats	Baicalin	Naturally occurring flavonoid non-specific RAGE modulator	↑Foetus body weight, ↑Placental weight, ↓VCAM– 1, ↓p65,↓ EGFR, ↓MCP–1, ↓NOX2, ↓RAGE, improvement of the animal metabolic prolife
2021	(Ref. 210)	Gestational diabetes mellitus – foetal development	Streptozotocin-induced diabetes mellitus rats	Ursolic acid	Naturally occurring alkaloid non-specific RAGE modulator	Improvement of the animal metabolic prolife, ↓ROS generation, ↓Risk of foetal development defects, ↓RAGE, ↓NOX2, ↓EGFR, ↓MCP–1
2016	(Ref. 224)	Gestational diabetes mellitus – foetal development	Streptozotocin-induced diabetes mellitus rats	RAGE knockout	Genetic modifications	↓Oxidative stress, ↓NF-κB activation, ↓Embryonic dysmorphogenesis
2017	(Ref. 223)	Intrauterine growth restriction (IUGR)	Mice exposed to second-hand smoke	Semi-synthetic glycosaminoglycan ethers (SAGEs)	Synthetic agents binding and blocking RAGE and RAGE ligands	Protected from foetal weight decrease, ↓TNF-α, ↓IL–1β, trophoblast invasive potential returned to normal levels
2023	(Ref. 252)	Preeclampsia	Bioinformatic analysis	Resveratrol	Naturally occurring polyphenol non-specific RAGE modulator	↓AGE-RAGE pathway, ↓IL6, ↓TNF, ↓IL1B,↓VEGFA, ↓STAT3, ↓EGFR
2020	(Ref. 264)	Placental vascular permeability	Bewo cell line	Ammonium pyrrolidinedithiocarbamate (PDTC), Anti-RAGE antibody	NF-κB inhibitor, antibody	Restored trans-epithelial electrical resistance (TEER), ↑ZO1, ↑Occludin, ↓Vascular permeability
2021	(Ref. 169)	Unexplained recurrent spontaneous abortion (URSA)	URSA mouse model	Aspirin	HMGB1 inhibitor	↓HMGB1, ↓TLR2, ↓TLR4, ↓NF-κB, ↓RAGE, ↓Expression of pyroptosis specific proteins, ↓Maternal–foetal interface destruction
2024	(Ref. 265)	Type I endometrial cancer	Bioinformatic analysis, mice model	Resveratrol	Naturally occurring polyphenol non-specific RAGE modulator	↑Phospho-ERK, ↓Hydroxylated oestrogen, restores oestrogen balance
2025	(Ref. 253)	Endometrial cancer	Bioinformatic analysis	Timosaponin A3	Naturally occurring alkaloid non-specific RAGE modulator	↓AGE-RAGE pathway, ↓Endometrial cancer progression, ↓Choline metabolism mediated by choline kinase alpha
2016	(Ref. 200)	Endometrial cancer	Mouse model xenografted with endometrial cancer cells	siRNA RAGE silencing	Genetic modifications	↓Proliferation rate, ↓Formation of microvessels and microvessel density
2019	(Ref. 202)	Endometrial cancer	Mouse model xenografted with endometrial cancer cells	Anti-RAGE antibody conjugated with antimitotic drugs	Conjugated antibody	↓Tumour volume, selective cytotoxicity for cancer cells
2021	(Ref. 255)	Ovarian cancer	Peritoneal mesothelial cells	Acacetin	Naturally occurring flavonoid non-specific RAGE modulator	↓Proliferation rate, ↓Invasion potential, ↑Apoptosis, ↓RAGE-PI3K/AKT signalling, ↓MMP2, ↓MMP9, ↓IL6, ↓IL8
2017	(Ref. 198)	Ovarian cancer	Ovarian cancer stem-like cells (CSLCs)	S100B knockdown	Genetic modifications	↓In-vitro self-renewal, ↓In-vivo tumourigenicity
2024	(Ref. 254)	Ovarian cancer	Bioinformatic analysis, A2780 ovarian cancer cell line	Sophorae flavescentis radix	Herbal medicine – non-specific RAGE modulator	↓Cancer cell viability
2020	(Ref. 256)	Cervical squamous cell carcinoma	Mouse model xenografted with cervical cancer cells	RAGE knockdown with lentivirus transfection, FPS-ZM1	Genetic modifications, RAGE inhibitor	↓Cancer cell proliferation, ↑Cancer cell apoptosis
2018	(Ref. 266)	Cervical cancer	Cervical squamous cancer cells	siRNA AGER silencing	Genetic modifications	↓AGER, ↓Apoptosis rate, ↓Migration potential
2023	(Ref. 257)	Triple negative breast cancer (TNBC)	TNBC mouse model	Azeliragon, FPS-ZM1	Drug, RAGE inhibitor	↓Metastatic potential, ↓Cellular adhesion, migration and invasive potential, ↓Pyk2, STAT3 and PI3K/Akt signalling pathways, ↓RAGE
2023	(Ref. 258)	Breast cancer	Bioinformatic analysis, breast cancer cell line	siRNA RAGE silencing, FPS-ZM1, RAGE antagonist peptide (RAP)	Genetic modifications, RAGE inhibitor, RAGE antagonist	↓Cancer cell proliferation, ↓Migration potential, ↓Patient-derived mammosphere formation

Abbreviations: AGE: advanced glycation end-products; Akt: protein kinase B; AMH: anti-müllerian hormone; AMHR-2: anti-müllerian hormone receptor type 2; BAX: BCL2-associated X protein; CRP: C-reactive protein; CYP19A1: cytochrome P450 family 19 subfamily A member 1/aromatase; E2: estradiol; EGFR: epidermal growth factor receptor; FOXO3a: forkhead box O3A; FSH: follicle-stimulating hormone; HbA1c: haemoglobin A1c; HDL: high-density lipoprotein; IL-1B: interleukin-1 beta; IL-6: interleukin-6; IL-8: interleukin-8; LDL: low-density lipoprotein; LH: luteinizing hormone; MMP2: matrix metalloproteinase-2; MMP9: matrix metalloproteinase-9; NF-κB p-p65: nuclear factor kappa-B phosphorylated p65 subunit; P4: progesterone; PCOS: polycystic ovary syndrome; PI3K: phosphoinositide 3-kinase; P53: tumour protein p53; RAGE: receptor for advanced glycation end-products, PI3K/Akt signalling: PI3K/Akt signalling pathway; ROS: reactive oxygen species; SMAD 1/5/8: SMAD family members 1, 5 and 8; STAR: steroidogenic acute regulatory protein; STAT3: signal transducer and activator of transcription 3; 17β-HSD: 17β-hydroxysteroid dehydrogenase; 3β-HSD: 3β-hydroxysteroid dehydrogenase; TLR4: Toll-like receptor 4; TNF-α: tumour necrosis factor-alpha; VCAM-1: vascular cell adhesion molecule-1; VEGF: vascular endothelial growth factor; VEGFA: vascular endothelial growth factor A; VLDL: very-low-density lipoprotein.

## RAGE receptor: Structure and isoform classification

**Figure 2.**
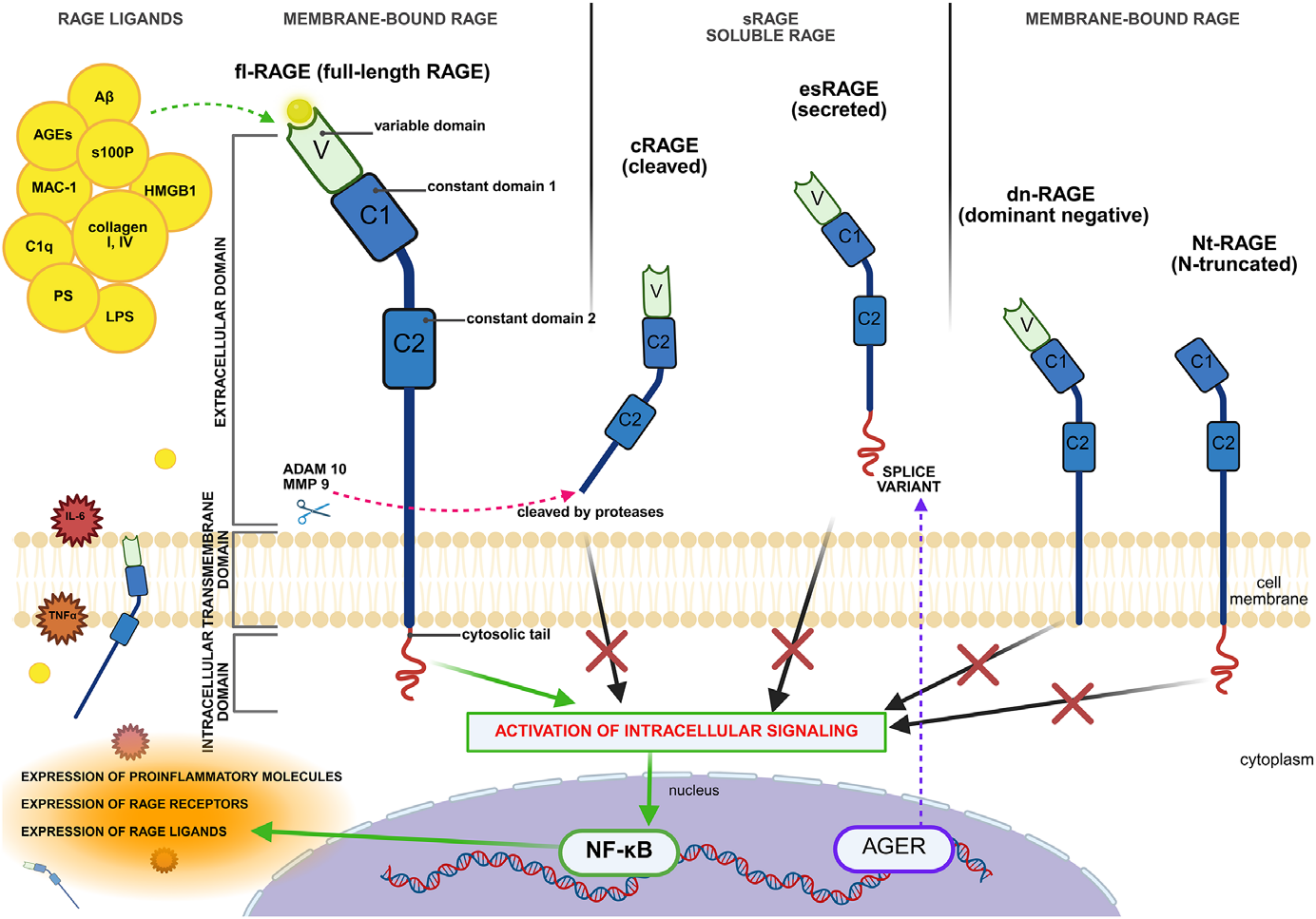
Structural organization of RAGE and its isoforms. The full-length RAGE (fl-RAGE) consists of three extracellular domains (V, C1 and C2), a transmembrane domain and a cytoplasmic tail essential for intracellular signalling. Soluble isoforms, including cleaved RAGE (cRAGE) and endogenous secretory RAGE (esRAGE), lack the transmembrane and cytoplasmic regions and act as decoy receptors. Other membrane-bound variants include dominant-negative RAGE (dn-RAGE), which interferes with signal transduction, and N-truncated RAGE (Nt-RAGE), which lacks the V domain required for ligand binding. Ligand binding to fl-RAGE activates intracellular pathways, such as NF-κB, leading to the expression of proinflammatory molecules, RAGE ligands and the AGER gene, thereby sustaining a positive feedback loop of inflammation. Abbreviations: Aβ: amyloid beta; ADAM10: a disintegrin and metalloproteinase 10; AGER: advanced glycation end-products receptor; AGEs: advanced glycation end-products; C1q: complement component 1q; cRAGE: cleaved receptor for advanced glycation end-products; dn-RAGE: dominant negative receptor for advanced glycation end-products; esRAGE: endogenous secretory receptor for advanced glycation end-products; fl-RAGE: full-length receptor for advanced glycation end-products; HMGB1: high mobility group box 1; IL6: interleukin 6; LPS: lipopolysaccharide; MAC-1: macrophage-1 antigen; MMP9: matrix metalloproteinase 9; NF-kβ: nuclear factor kappa beta; NT-RAGE: N-terminal receptor for advanced glycation end-products; sRAGE: soluble receptor for advanced glycation end-products; TNF: tumour necrosis factor. Created in BioRender. Łuszczyński, K. (2026) https://BioRender.com/aqlvelc. Figure 2. long description.

RAGE is composed of three main regions: an intracellular tail, a transmembrane helix and an extracellular domain (Ref. 32). The extracellular domain consists of three immunoglobulin-like subdomains: two constant (C) subdomains, C1 and C2, and one variable (V) subdomain. The V and C1 domains form an integrated VC1 ectodomain (via amino acids linkers), which is involved in the interaction with a wide range of RAGE ligands (Refs 33, 34). In addition to the membrane-bound full-length RAGE (fl-RAGE), several other isoforms exist, characterized by the presence or absence of specific domains. These isoforms can be broadly categorized into two main groups: soluble and membrane-bound variants with altered signalling properties. The diversity of RAGE isoforms is presented in Figure 2.

The first group includes two major soluble forms of the receptor, both lacking transmembrane and cytoplasmic domains. These isoforms can be detected in blood or other bodily fluids and are collectively referred to as soluble RAGE (sRAGE). In contrast to membrane-bound RAGE, sRAGE lacks signalling capacity and instead acts as a decoy receptor by binding RAGE ligands. This interaction prevents the ligands from binding to membrane-bound RAGE, thereby suppressing subsequent RAGE-mediated signalling cascades (Ref. 32).

Within soluble isoforms, cleaved RAGE (cRAGE), formed by proteolytic cleavage between the extracellular and transmembrane domains of RAGE by two metalloproteinases MMP9 and ADAM10, can be identified (Ref. 35). This cleavage is stimulated by inflammatory signals, including HMGB1, lipopolysaccharides (LPS) or TNF-α. Moreover, RAGE overexpression promotes the activity of both matrix metalloproteinase-9 (MMP9) and a disintegrin and metalloproteinase domain-containing protein 10 (ADAM10), thereby enhancing proteolytic cleavage of RAGE and serving as a negative feedback mechanism regulating RAGE overexpression (Ref. 36). Other soluble form is endogenous secretory RAGE (esRAGE or RAGEv1), which origin from alternative splicing of RAGE pre-mRNA (Ref. 37). Although the precise mechanism regulating esRAGE formation remains unclear, studies in neuroblastoma cell lines have shown that glucose deprivation leads to reduced esRAGE expression and an increased RAGE/esRAGE ratio. This suggests that disrupted glucose metabolism may influence esRAGE expression levels (Ref. 38).

The second category of isoforms includes membrane-bound RAGE proteins lacking specific functional domains, resulting in altered or absent signalling capability. One such variant is dominant-negative RAGE (dnRAGE), a membrane-bound isoform that lacks the cytoplasmic domain required for signal transduction. As a result, it cannot initiate intracellular signalling but may still bind RAGE ligands, serving as a decoy receptor and modulating RAGE-mediated inflammatory pathways (Ref. 39). However, the biological effects of dnRAGE appear to differ according to the disease. For instance, Takeuchi et al. showed that its overexpression in a human fibrosarcoma cell line significantly decreased the proliferation, migration and invasive potential of the tumour cells (Ref. 40). In contrast, in lung adenocarcinoma, dnRAGE is the predominant RAGE phenotype, and evidence suggests that it promotes epithelial–mesenchymal transition, enhances cellular motility and facilitates metastasis. Moreover, it may also suppress or reverse fl-RAGE expression, although the exact molecular mechanism remains unknown (Ref. 41).

Another membrane-bound variant is the N-truncated isoform of RAGE (N-RAGE), which lacks the N-terminal V domain required for receptor–ligand interactions. As a result, it is unable to bind AGEs. It can still be activated, however, via a mechanism that does not depend on the V domain, but the exact physiological role and mode of activation of N-RAGE remain to be determined (Ref. 42). Additionally, RAGE isoform diversity arises not only through alternative splicing (Ref. 37) and post-translational modifications (Ref. 43) but also through genetic polymorphisms that may affect receptor function (Ref. 44).

Advanced molecular biology techniques, such as RNA sequencing (RNA-seq) (Ref. 45), quantitative reverse transcription PCR (qRT-PCR) using isoform-specific primers (Ref. 46), Western blotting with antibodies targeting unique epitopes (Ref. 47) and proteomic approaches (Ref. 44), could be employed in future studies to distinguish between these isoforms (Ref. 48).

However, even though the detailed investigation and differentiation among various isoforms and post-translational modifications of RAGE is pivotal in deciphering and understanding molecular mechanisms driving diseases on cellular level. Due to high level of interactions complexity and similarity between molecules sometimes researchers focused on the most abundant isoforms or did not specify which group of isoform they studied and described them collectively as RAGE. Therefore, everywhere where it was possible, we presented the most detailed isoform-specific descriptions and analyses possible.

## RAGE ligands and RAGE-mediated signalling pathways

RAGE is activated by a broad spectrum of structurally and functionally diverse ligands. It was initially characterized for its ability to bind advanced glycation end-products (AGEs) (Ref. 49) – a heterogeneous group of molecules formed via non-enzymatic glycation of proteins, lipids or nucleic acids, a process accelerated by factors such as cigarette smoke exposure, high-calorie diets rich in refined carbohydrates and a sedentary lifestyle (Ref. 50). Glycation alters the structural properties of proteins and their receptor-binding capacity; for instance, glucose bovine serum albumin (glucose-BSA) and fructose bovine serum albumin (fructose-BSA) exhibit stronger binding to RAGE than highly cross-linked ribose bovine serum albumin (ribose-BSA) (Ref. 51). Moreover, specific glycation sites can significantly influence ligand behaviour. Notably, glycation of RAGE itself reduces binding affinity for glucose-BSA and completely abolishes interaction with fructose-BSA (Ref. 51). These findings highlight glycation-dependent modulation of ligand–receptor interactions as a potential therapeutic target to disrupt RAGE-mediated signalling.

Recent studies have characterized RAGE as a pattern recognition receptor (PRR), analogous to Toll-like receptors (TLRs). Interestingly, RAGE identifies the three-dimensional conformations of its ligands rather than specific amino acid sequences. This structural flexibility enables it to bind a wide range of compounds that lack sequence homology, including various endogenous danger-associated molecular patterns (DAMPs) released by damaged or stressed cells, as well as pathogen-associated molecular patterns (PAMPs) derived from infectious microorganisms or environmental sources (Refs 51, 52). Other RAGE ligands can be classified into two groups: exogenous and endogenous. Exogenous ligands comprise a highly heterogeneous array of molecules derived from pathogens and foreign substances (Ref. 15), while endogenous ligands are produced within the body and include small α-helical proteins such as high-mobility group box 1 (HMGB1) (Ref. 3), members of the S100 protein family (Ref. 4), protein oligomers and aggregates such as β-amyloid, collagen types I and IV (Ref. 5), transmembrane proteins such as MAC-1 (Ref. 6), nucleic acids (Ref. 53), complement system proteins including complement component 1q (C1q) (Ref. 7), and apoptosis-associated markers such as phosphatidylserine (PS) (Ref. 54). The binding of RAGE to its ligand initiates an immediate cellular response. This process may be influenced not only by changes in membrane fluidity or receptor glycation (e.g., during ageing) but also by the stability of the RAGE–ligand complex, particularly the duration of its dissociation phase (Ref. 51).

One of the most potent ligands of RAGE is high-mobility group box 1 (HMGB1), also known as amphoterin, which was the first non-AGE ligand identified for this receptor. Detailed studies of the amphoterin–RAGE interaction demonstrated that the binding is specific, saturable and of higher affinity than that observed for AGEs (Ref. 55). Based on its dissociation constant (Kd), a parameter commonly used to assess ligand–receptor affinity, HMGB1 exhibits one of the highest binding affinities among known RAGE ligands, with a Kd in the range of 6–10 nM (Ref. 33). Interestingly, HMGB1 interacts not only with RAGE but also with several other cell surface receptors, among which RAGE and Toll-like receptor 4 (TLR4) are among the most extensively studied (Ref. 1). Under homeostatic conditions, HMGB1 is primarily localized in the nucleus, where it functions as a DNA chaperone, modulates gene transcription and participates in the recruitment of transcription factors. Upon cellular damage or during certain forms of cell death, HMGB1 is passively released into the extracellular space, acting as a DAMP and triggering an inflammatory response (Ref. 1). Additionally, it can be actively secreted following stimulation by various exogenous microbial products (e.g., lipopolysaccharide, LPS) or proinflammatory cytokines such as tumour necrosis factor-α (TNF-α) and interleukin-1 (IL-1) (Ref. 56). Interestingly, naturally elevated levels of HMGB1 in menstrual blood may contribute to the formation of endometriotic lesions following retrograde menstruation by promoting inflammation and angiogenesis (Ref. 57). Furthermore, HMGB1 has also been proposed as a potential biomarker for endometriosis (Ref. 58).

Other potent ligands of RAGE are proteins from the S100 family, a group of small (molecular weight of 9–14 kDa) calcium-binding proteins expressed exclusively in vertebrates (Ref. 59). Under physiological conditions, S100 proteins function as calcium sensors, mediating calcium-dependent signalling through interactions with specific target proteins. They regulate a variety of cellular processes, including gene expression, cell cycle progression and inflammatory responses (Ref. 60). In humans, more than 20 S100 isoforms are encoded within the chromosome 1q21 locus, known as the epidermal differentiation complex (EDC) cluster (Ref. 59). S100 protein expression is often tissue-specific; for instance, S100A1 is predominantly found in cardiac myocytes (Ref. 61), while S100A3 is primarily expressed in hair follicle cells (Ref. 62). Upon cellular stress or damage, S100 proteins are released into the extracellular space, where they act as DAMPs, triggering the production of proinflammatory cytokines through interactions with pattern recognition receptors such as RAGE (Ref. 63). Several members of the S100 family have been identified as RAGE ligands, including S100A1, S100A2, S100A4 (metastasin), S100A5, S100A6 (calcyclin), S100A7 (psoriasin 1), S100A8 (calgranulin A), S100A9 (calgranulin B), the S100A8/A9 heterodimer (calprotectin), S100A11 (calgizzarin), S100A12 (calgranulin C), S100A13, S100A14, S100B and S100P (Ref. 60). Among them, one of the most potent ligands is S100A12, which binds to the C1 domain of RAGE with a dissociation constant (Kd) of approximately 70 nM (Ref. 33). Notably, S100A12 – also known as extracellular newly identified receptor for advanced glycation end-products binding protein (EN-RAGE) – has been found at significantly elevated levels in individuals with endometriosis (Ref. 64). Emerging evidence suggests that chronic inflammation in endometriosis may be driven by the RAGE–EN-RAGE signalling axis (Ref. 24). Additionally, activation of RAGE by its ligand, the S100A8/A9 heterodimer, has been shown to promote fibrotic remodelling within endometrial tissue (Ref. 65).

Another well-characterized RAGE ligand, with a high binding affinity, is amyloid β (Aβ), which contributes to oxidative stress and the upregulation of proinflammatory cytokine gene expression in neural tissue through microglial activation (Ref. 66). RAGE also binds various endogenous danger signals, including extracellular RNA and DNA. When complexed with RAGE, these nucleic acids may promote inflammasome activation and enhance the expression of interleukin-1β (Ref. 67). In addition, RAGE engages with several substances regarded as PAMPs, such as lipopolysaccharide (LPS), highlighting its key role in modulating innate immune responses (Ref. 52).

Within the plasma membrane, RAGE predominantly exists as preassembled dimers or higher-order multimers, which undergo further oligomerization upon stimulation by RAGE ligands (Ref. 68). This process increases the number of available binding sites and precedes both ligand binding and signal transduction (Ref. 69). Ligand-induced oligomerization induces conformational changes in the receptor’s cytoplasmic tail, enabling the recruitment of adaptor proteins such as diaphanous-related formin 1 (DIAPH1) and Toll/interleukin-1 receptor domain-containing adaptor protein (TIRAP), which in turn activate multiple intracellular signalling cascades (Ref. 70).

DIAPH1 engages members of the Rho GTPase family, including cell division control protein 42 (Cdc42) and Ras-related C3 botulinum toxin substrate 1 (Rac1), thereby promoting the activation of NF-κB through the p38 MAPK pathway. NF-κB can also be activated via the canonical MAPK cascade involving Ras GTPase and extracellular signal-regulated kinases 1 and 2 (ERK1/2) signalling (Ref. 71). Moreover, DIAPH1 has been shown to stimulate interferon-stimulated response elements (ISRE), further amplifying the inflammatory response (Ref. 72).

**Figure 3.**
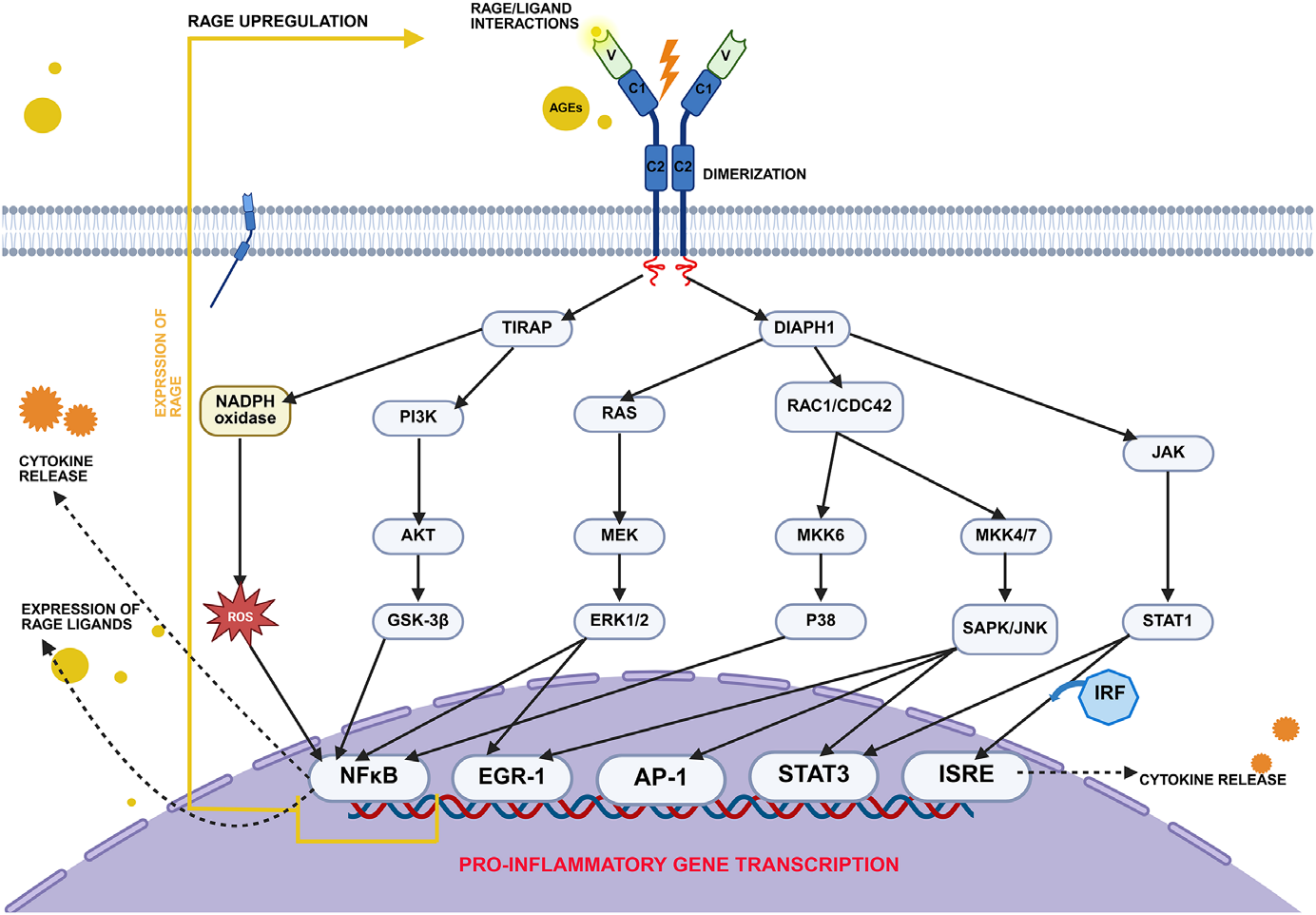
RAGE-mediated intracellular signalling pathways. Upon ligand binding, RAGE undergoes oligomerization and activates multiple intracellular signalling cascades via adaptor proteins such as DIAPH1 and TIRAP. These include the MAPK (ERK1/2, p38, JNK), PI3K/AKT and JAK/STAT pathways, as well as ROS generation via NADPH oxidase. Downstream activation of transcription factors – including NF-κB, AP-1, EGR-1, STAT1/3 and IRFs – leads to the expression of proinflammatory genes. This results in cytokine production and upregulation of both RAGE and its ligands, creating a positive feedback loop that sustains chronic inflammation. Abbreviations: AKT: protein kinase B; AP-1: activator protein 1; DIAPH1: diaphanous-related formin 1; EGR-1: early growth response 1; ERK1/2: extracellular signal-regulated kinases 1 and 2; GSK-3B: glycogen synthase kinase 3 beta; IRF: interferon regulatory factor; ISRE: interferon-stimulated response element; JAK: Janus kinase; MEK: mitogen-activated protein kinase kinase; MKK4/7: mitogen-activated protein kinase kinase 4 and 7; MKK6: mitogen-activated protein kinase kinase 6; NADPH oxidase: nicotinamide adenine dinucleotide phosphate oxidase; NFKB: nuclear factor kappa B; P38: p38 mitogen-activated protein kinase; PI3K: phosphoinositide 3-kinase; RAC1/CDC42: Ras-related C3 botulinum toxin substrate 1 and cell division control protein 42; RAS: rat sarcoma virus oncogene; SAPK/JNK: stress-activated protein kinase c-Jun N-terminal kinase; STAT1: signal transducer and activator of transcription 1; STAT3: signal transducer and activator of transcription 3; TIRAP: Toll interleukin-1 receptor domain-containing adaptor protein. Created in BioRender. Łuszczyński, K. (2026) https://BioRender.com/8niuuhk. Figure 3. long description.

In parallel, TIRAP facilitates AKT phosphorylation and plays an essential role in initiating the NF-κB signalling pathway (Ref. 73). The RAGE-mediated signalling pathways are presented in Figure 3.

## RAGE in health: Expression patterns and functional roles in the female reproductive tract

In most mature tissues, excluding the lungs, RAGE is expressed at low or undetectable levels under normal physiological conditions (Ref. 13). However, emerging evidence suggests that RAGE-mediated signalling may play a role in several physiological processes related to female reproductive function and pregnancy.

Bhutada et al. demonstrated that the concentration of RAGE ligands in the endometrium fluctuates throughout the menstrual cycle and is significantly lower during the receptive phase, suggesting a regulatory role in endometrial receptivity (Ref. 74). RAGE expression has also been identified in maternal tissues directly involved in pregnancy, such as chorionic villi (Ref. 75), as well as in tissues indirectly involved, including the myometrium and omental fat (Ref. 76).

Choltus et al. observed increased expression of RAGE and its ligand HMGB1 in foetal membrane zones with altered morphology that are more susceptible to rupture. These findings suggest a role for RAGE in promoting sterile inflammation that contributes to membrane weakening during labour (Ref. 77). This is further supported by Colte et al., who reported significantly higher RAGE expression in the amnion and in primary amnion-derived epithelial cells during spontaneous term labour compared to non-labouring conditions (Ref. 78).

HMGB1 levels were also found to be elevated in umbilical cord venous blood during both spontaneous and induced labour when compared to non-labouring women. Moreover, spontaneous vaginal deliveries were associated with significantly higher HMGB1 levels than elective or emergency caesarean sections, suggesting a potential role for this ligand in labour initiation and progression depending on the mode of delivery (Ref. 79).

In addition to human studies, functional investigations in animal models have shown that RAGE and its ligands may be involved in key developmental processes such as decidualization (Ref. 80), blastocyst implantation (Ref. 81), neurulation (Ref. 82) and embryonic limb development (Ref. 83).

Although these findings offer promising insights into the physiological involvement of RAGE, the current research in this area remains limited in scope. Most available studies have focused on a narrow set of maternal or foetal tissues and often rely on semi-quantitative methods such as immunohistochemistry or Western blotting, with only a few employing high-throughput or functional approaches. Additionally, sample sizes across studies tend to be modest, and temporal aspects of RAGE signalling remain poorly defined. As summarized in Table 1, further large-scale and mechanistic studies are required to clarify the regulatory role of RAGE in the female reproductive tract and its physiological relevance to pregnancy-associated processes.

## The role of RAGE in benign gynaecological disorders

### RAGE in benign endometrial disorders – endometriosis and intrauterine adhesions

Within the spectrum of benign endometrial disorders, the currently available literature on RAGE is limited primarily to endometriosis and intrauterine adhesions. This restricted evidence base highlights the need for further studies to clarify the role of RAGE in other non-malignant endometrial pathologies, such as endometrial polyps or hyperplasia. Endometriosis is one of the most common benign endometrial disorders affecting approximately 10% of women of reproductive age and characterized by the presence of endometrial-like tissue outside the uterine cavity and clinically associated with dysmenorrhea, chronic pelvic pain, dyspareunia and infertility (Ref. 84).

Several studies have demonstrated that RAGE expression is increased in the endometrium of patients with endometriosis. Moreover, the RAGE–EN-RAGE axis has been implicated in sustaining the chronic inflammation underlying this condition (Ref. 24). Among RAGE ligands, HMGB1 has also been proposed as a potential biomarker for the diagnosis of endometriosis (Ref. 58). It has been suggested that the naturally elevated levels of HMGB1 in menstrual blood, which may reach the peritoneal cavity through retrograde menstruation, contribute to lesion formation by promoting inflammation and angiogenesis (Ref. 57). Similarly, increased concentrations of EN-RAGE (S100A12) and carboxymethyl lysine (CML), another well-established RAGE ligand, have also been reported in women with endometriosis (Refs 64, 85).

Fujii et al. reported that the concentration of soluble RAGE (sRAGE) is significantly elevated in the follicular fluid of patients with endometriosis (Ref. 85). Interestingly, subsequent studies have shown that sRAGE levels positively correlate with the number of retrieved oocytes and the number of good-quality embryos, suggesting that sRAGE may serve as a positive predictive marker for in vitro fertilization (IVF) outcomes in these patients (Ref. 86).

Despite these promising findings, the available research on RAGE involvement in endometriosis remains relatively limited, as presented in Table 2. Most published studies are characterized by small sample sizes, variable inclusion criteria and heterogeneity in tissue sources (e.g., follicular fluid, peritoneal fluid, plasma, eutopic and ectopic endometrium). Additionally, the methodological approaches are inconsistent across studies, with ELISA and qRT-PCR being most commonly employed, while advanced omics techniques such as RNA sequencing are rarely used. These limitations underscore the need for larger, standardized and mechanistically oriented studies to validate the diagnostic or prognostic potential of RAGE-associated molecules in endometrial pathologies.

Transcriptomic analysis of scar tissue from patients with endometrial fibrosis has revealed an upregulation of genes associated with the AGE–RAGE signalling pathway (Ref. 87). Furthermore, stimulation of RAGE by its ligand S100A8/A9 has been shown to promote intrauterine adhesion formation, highlighting the role of RAGE-mediated signalling in fibrotic remodelling of the endometrium (Ref. 65).

### Pathologies of ovaries – polycystic ovary syndrome

Polycystic ovary syndrome (PCOS) is one of the most common endocrine disorders in women of reproductive age. It is a complex condition characterized by coexisting metabolic dysregulation and reproductive dysfunction, including anovulation and infertility, hormonal imbalance with hyperandrogenism, insulin resistance and often obesity (Ref. 90).

Recent findings suggest that RAGE-mediated signalling may play a role in both the metabolic and reproductive aspects of PCOS (Table 3). Multiple studies have demonstrated significantly reduced levels of the protective isoform sRAGE in follicular fluid from PCOS patients (Refs 91, 92, 93), although in one study no difference in sRAGE concentrations between PCOS patients and controls was reported (Ref. 94).

In terms of local tissue involvement, immunostaining of ovarian biopsies from PCOS patients revealed altered distribution of AGEs, with increased accumulation in endothelial cells and ovarian granulosa cells. These cells also exhibited enhanced expression of RAGE and exclusive activation of the NF-κB p65 subunit, suggesting persistent activation of inflammatory signalling pathways (Ref. 25). Further supporting this, granulosa-lutein cell lines derived from PCOS patients showed increased expression of RAGE and its ligands S100A9 and AGEs (Refs 95, 96). Notably, exposure of these cells to AGEs resulted in upregulation of the Anti-Müllerian Hormone (AMH) receptor, which is known to inhibit folliculogenesis by preventing the maturation and atresia of early-stage follicles.

Functional studies in both clinical and preclinical models have highlighted the impact of RAGE signalling on key features of PCOS, such as infertility, hyperandrogenism and insulin resistance. In animal models, accumulation of AGEs in ovarian tissue was associated with dysregulated extracellular matrix (ECM) organization and overexpression of lysyl oxidase (LOX), contributing to increased stromal density and potential cyst formation (Ref. 97). Additionally, a diet high in AGEs led to reduced activity of glyoxalase I (GLO-I) in the ovary, an enzyme responsible for detoxifying reactive carbonyl species (Ref. 98).

There is also evidence linking AGE–RAGE signalling with hormonal dysregulation in PCOS. In both rats (Ref. 99) and humans (Ref. 100), dietary AGE intake was associated with increased serum testosterone levels. Moreover, Chatzigeorgiou et al. demonstrated that increased AGE consumption reduced estradiol and progesterone levels in female rats (Ref. 101). Further, Azhary et al. showed that testosterone stimulation itself can increase RAGE expression and AGE accumulation in granulosa-lutein cells via endoplasmic reticulum stress (Ref. 96). The role of RAGE in insulin resistance in PCOS has also been explored. Notably, women who adhered to a diet low in AGEs demonstrated improved insulin sensitivity, independent of changes in body weight or insulin secretion (Ref. 102).

Despite these compelling findings, the current studies examining RAGE in PCOS remain limited and heterogeneous. While several publications consistently report altered RAGE expression and sRAGE deficiency in PCOS, variability in sample sizes, methodological differences and inconsistent findings (particularly regarding sRAGE levels in serum vs. follicular fluid) underscore the need for further validation. Additionally, although one large-scale study found no significant association between RAGE gene polymorphisms (-429T>C and -374T>A) and PCOS (Ref. 103), altered post-translational regulation and tissue-specific dynamics of RAGE may still be clinically relevant. Therefore, existing data support the involvement of RAGE and its ligands in the inflammatory, hormonal and metabolic dysregulation characteristic of PCOS. However, further high-quality, mechanistic studies are warranted to clarify the functional implications of RAGE signalling and to determine its potential utility as a diagnostic biomarker or therapeutic target in this condition.

### Pregnancy-related pathologies: Hypertensive disorders

Hypertension complicates 5%–10% of all pregnancies and remains a leading cause of maternal and foetal morbidity and mortality worldwide. Hypertensive disorders in pregnancy (HDP), which include chronic hypertension, gestational hypertension, preeclampsia (PE) and preeclampsia superimposed on chronic hypertension, are all associated with an elevated risk of long-term maternal cardiovascular and cerebrovascular complications. Among these, preeclampsia carries the highest risk of multiorgan failure and disseminated intravascular coagulation (DIC). Foetal risks include intrauterine growth restriction (IUGR), preterm birth and stillbirth, with the highest incidence in preeclampsia cases (Refs 108, 109).

Preeclampsia is clinically defined by the onset of hypertension after the 20th week of gestation, accompanied by signs of end-organ dysfunction, such as proteinuria, visual disturbances or HELLP (haemolysis, elevated liver enzymes and low platelets) syndrome. On a molecular level, insufficient trophoblast invasion and inadequate spiral artery remodelling may lead to placental hypoperfusion, hypoxia and oxidative stress, ultimately triggering inflammation (Ref. 110). Among the implicated inflammatory mediators, damage-associated molecular patterns (DAMPs), potent RAGE ligands, have been proposed as key contributors to the sterile inflammation observed in PE (Ref. 111).

Numerous studies have investigated the role of RAGE signalling in preeclampsia (Table 4). Collectively, these data suggest a widespread upregulation of RAGE and its ligands across tissues and fluids involved in the disease. One group of studies focused on placental and foetal membrane samples due to their direct involvement in PE pathogenesis. These studies consistently demonstrated increased RAGE and HMGB1 expression at both mRNA and protein levels (Refs 26, 112, 113, 114, 115, 116). However, Schwertner et al. (Ref. 48) reported undetectable RAGE levels in some PE placentae, attributing this to antibody limitations, thereby underscoring the importance of method selection in RAGE research.

Another group of studies evaluated systemic changes in RAGE and its ligands using peripheral blood, umbilical cord blood and amniotic fluid samples. These analyses revealed increased concentrations of RAGE, AGEs, HMGB1 and S100 proteins, with the strongest changes observed in severe PE or PE complicated by acute heart failure (Refs 117, 118, 119). Interestingly, while membrane-bound RAGE expression was elevated, levels of its soluble isoforms (sRAGE and esRAGE) were also increased in several studies (Refs 120, 121, 122, 123). This paradoxical finding may reflect a compensatory response through alternative splicing or proteolytic cleavage aimed at counteracting excessive RAGE activation (Ref. 124).

Functional studies further support the pathological role of RAGE signalling in PE. Akasaka et al. (Ref. 125) showed that human adipocytes incubated with serum from PE patients exhibited increased interleukin 6 (IL-6) and C-C motif chemokine ligand 2 (CCL2) expression, which was significantly reduced following RAGE silencing via small interfering RNA (siRNA). Similarly, Zenerino et al. (Ref. 112) demonstrated that low molecular weight heparin attenuates RAGE-mediated inflammation in preeclampsia-derived tissue explants by neutralizing HMGB1.

### Pregnancy-related pathologies: Metabolic diseases

During pregnancy, the maternal body undergoes extensive physiological changes to support foetal development, including numerous metabolic adaptations. Among them, hormonal alterations lead to a physiologic reduction in insulin sensitivity and a slight elevation in mean blood glucose levels to meet the metabolic demands of the growing foetus (Ref. 130). However, in some individuals, these adaptations are insufficient, resulting in metabolic disorders that impact both maternal and foetal health. The most prevalent conditions include gestational diabetes mellitus (GDM) and pre-existing diabetes mellitus (type 1 or type 2).

GDM is one of the most common metabolic complications of pregnancy. It typically develops during the second or third trimester and affects up to 14% of pregnancies worldwide. It is characterized by glucose intolerance not present before pregnancy and usually resolves after delivery (Ref. 131). Risk factors include advanced maternal age, excessive gestational weight gain, pre-pregnancy overweight or obesity and underlying conditions associated with insulin resistance, such as polycystic ovary syndrome (PCOS) (Ref. 132).

Given the well-established role of the receptor for advanced glycation end-products (RAGE) in glucose metabolism and diabetic complications, several studies have investigated its involvement in GDM and other pregnancy-related metabolic disorders. These studies revealed altered expression of RAGE and its ligands across various maternal and foetal compartments, although the findings are not entirely consistent (Table 5). For instance, in peripheral blood, a consistent increase in RAGE and its ligands, especially advanced glycation end-products (AGEs) such as Nε-(carboxymethyl)lysine (CML), has been observed, with levels correlating with disease severity (Ref. 133). Similarly, elevated *RAGE* expression was confirmed in maternal omental adipose tissue and endometrial samples, particularly in obese pregnant women (Refs 134, 135). Additionally, increased expression of HMGB1, a pro-inflammatory RAGE ligand, was found in foetal membranes of women with GDM (Ref. 134), while umbilical cord tissue and human umbilical vein endothelial cells (HUVECs) derived from diabetic pregnancies exhibited upregulation of RAGE and AGEs (Refs 136, 137).

In contrast, levels of soluble RAGE (sRAGE) were found to be decreased in women with type 1 diabetes during pregnancy (Ref. 138), which may suggest impaired regulatory mechanisms. These findings suggest a potential contribution of the AGE–RAGE axis to diabetes-associated vascular inflammation. Surprisingly, some studies found no significant differences in *RAGE* expression between GDM and control groups (Refs 48, 113), while others reported a reduction in RAGE mRNA and protein levels, possibly due to placental adaptation or glucose-buffering mechanisms (Ref. 116). It has been speculated that these discrepancies may also result from interventional factors such as insulin therapy or dietary management, which could mitigate hyperglycaemia-induced upregulation of *RAGE* (Refs 116, 139). Also, although polymorphisms in the *RAGE* gene have been linked to metabolic disease risk in other contexts, large-scale genotyping studies have not confirmed a significant association between -429T>C and -374T>A polymorphisms and GDM (Ref. 140).

Collectively, current data suggest that the RAGE signalling pathway is activated in several tissues in response to gestational metabolic imbalance; however, its role may differ depending on tissue type, disease severity or treatment status. Further mechanistic and longitudinal studies are required to elucidate the exact role of RAGE in hyperglycaemia-related pregnancy complications.

### Pregnancy-related pathologies: Pathologies of foetal membranes and premature birth

Foetal membranes play a critical role in maintaining a healthy pregnancy by regulating amniotic fluid homeostasis and serving as both physical and immunological barriers that protect the developing foetus. Structurally, they consist of two distinct layers: the amnion, the innermost layer in direct contact with the amniotic fluid, and the chorion, which lies adjacent to the maternal decidua (Ref. 142). Towards the end of gestation, a tightly regulated weakening of the foetal membranes occurs through processes such as inflammation, cellular senescence and apoptosis, ultimately leading to rupture of membranes (ROM) that is a key step in the initiation of labour. Recent research has highlighted the role of sterile inflammation, a non-infectious inflammatory response, in regulating this process. This inflammation is mediated by the activation of pattern recognition receptors (PRRs) in response to DAMPs, many of which are present in the amniotic fluid (Ref. 143).

Premature activation of these inflammatory pathways can result in preterm premature rupture of membranes (pPROM), which affects approximately 3%–4% of pregnancies and accounts for more than 40% of all preterm births. pPROM is associated with elevated perinatal morbidity, mortality and long-term complications, underscoring the need to better understand the molecular mechanisms driving foetal membrane rupture (Ref. 144). Although the exact molecular mechanisms remain incompletely understood, RAGE, as a key receptor for multiple DAMPs, has been implicated as a central player in foetal membrane pathology.

In this context, several studies have explored the role of RAGE signalling (Table 6). Yan et al. reported increased expression of RAGE and its ligand HMGB1 in both placental tissue and peripheral blood of women with pPROM, a pattern not observed in premature rupture of membranes (PROM) cases at term (Ref. 145). In another study, Rzepka et al. observed significantly elevated esRAGE levels in patients with pPROM, while sRAGE levels were not significantly different. Notably, sRAGE concentrations positively correlated with gestational age at delivery and neonatal birth weight, suggesting a modulatory rather than a pathogenic role (Ref. 146). Consistently, EN-RAGE (S100A12) was elevated in amniotic fluid during threatened preterm labour, further implicating RAGE ligands in foetal membrane weakening (Ref. 147).

Another area of investigation was focused on chorioamnionitis, also known as intrauterine inflammation (IUI), which is a frequent infectious complication of pregnancy, characterized by inflammation of the foetal membranes due to ascending bacterial infections. Clinical signs include maternal fever, uterine tenderness, leukocytosis and abnormal vaginal discharge (Ref. 148). Multiple studies report elevated concentrations of HMGB1, EN-RAGE, S100A8/A9 and S100A8 in amniotic fluid and placental tissues (Refs 149, 150, 151). However, findings regarding the soluble isoforms of RAGE are inconsistent. While Romera et al. observed increased levels of both sRAGE and esRAGE in chorioamnionitis (Ref. 152), other reports found decreased sRAGE, particularly in cases with culture-positive chorioamnionitis (Ref. 151). Overall, elevated tissue expression of RAGE and its ligands consistently correlated with disease severity and supports the hypothesis of RAGE-mediated amplification of the intrauterine inflammatory response.

A third major topic of analysis, that is, spontaneous preterm labour (sPTL), defined as regular uterine contractions with cervical changes occurring between 20 and 37 weeks of gestation, has also been linked to RAGE dysregulation. Several studies report increased concentrations of RAGE ligands, particularly EN-RAGE and HMGB1, in maternal serum, foetal membranes and amniotic fluid (Refs 153, 154, 155). In maternal blood, sRAGE levels were generally decreased, suggesting impaired anti-inflammatory buffering capabilities. Conversely, in threatened preterm labour (TPL), defined by uterine contractions without cervical dilation, Hajek et al. found significantly elevated sRAGE levels in maternal serum, which may potentially indicate a compensatory response (Ref. 156). In contrast, other studies using broader inclusion criteria (including pPROM) reported reduced sRAGE in TPL (Ref. 157), possibly reflecting underlying sterile inflammation.

Finally, environmental and infectious factors have also been examined. Choltus et al. demonstrated that exposure of amniotic epithelial cells and foetal membrane explants to cigarette smoke extract upregulated HMGB1 and gelatinase activity, implicating cigarette smoke in RAGE-mediated sterile inflammation and membrane weakening (Ref. 158). In the context of viral infection, Borges-Vélez et al. reported elevated total RAGE expression in placental tissue from Zika virus-infected pregnancies but reduced RAGE expression specifically in trophoblast cells. This cell-specific downregulation of RAGE may aid in viral immune evasion, allowing viral persistence inside the trophoblast layers. These findings imply that regulating RAGE activation in the placenta may be crucial in preventing vertical infection transmission and maintaining placental immunological balance (Ref. 159).

### Pregnancy-related pathologies: Pregnancy loss

Recurrent spontaneous abortion (RSA) is defined by the American Society for Reproductive Medicine as the occurrence of two or more consecutive pregnancy losses before 20 weeks of gestation with the same partner. It is estimated to affect approximately 1% of all pregnancies. Although several causative factors, such as immunological, chromosomal or structural abnormalities, have been identified, in about 50% of cases, the exact aetiology remains elusive (Ref. 165). Therefore, additional research is needed to further elucidate the pathophysiology of this distressing condition.

As RAGE involvement in decidualization and embryonic development has been well documented in animal models, this receptor appears to be a promising target for further investigation (Table 7). Xiao et al. demonstrated that RAGE expression is decreased in trophoblast tissue from patients experiencing RSA (Ref. 166), while Wu et al. reported similar findings in peripheral blood samples (Ref. 167). Given the essential role of RAGE in early pregnancy events, both before and during blastocyst implantation, insufficient expression or activation of this receptor may impair proper decidualization, ultimately leading to pregnancy loss. Additionally, studies by Zhu et al. and Zou et al. have shown that excessively high levels of HMGB1 during pregnancy may trigger chronic inflammation, resulting in tissue damage and disruption of the maternal–foetal interface. This hypothesis is further supported by evidence that aspirin, a drug known to reduce extracellular HMGB1 levels, can effectively lower the risk of RSA (Refs 168, 169). Finally, research by Ota et al. revealed that sRAGE levels are increased in patients with RSA, suggesting its potential role as a biomarker of RAGE-mediated uterine blood flow restriction and subsequent foetal ischaemia (Ref. 170).

### Pregnancy-related pathologies: Infant morbidities

Given the pivotal role of RAGE in physiological pregnancy and embryonic development, several studies have investigated its potential involvement in neonatal and infant morbidities. These efforts aimed to determine whether dysregulation of RAGE signalling contributes to adverse outcomes during early life.

Intrauterine growth restriction (IUGR) is defined as a condition in which the foetus fails to reach its genetically determined growth potential and exhibits clinical features of malnutrition or impaired intrauterine development. Epidemiologically, IUGR is most prevalent in developing regions, with Asia accounting for approximately 70% of global cases. Multiple contributing factors have been identified, including maternal, foetal and placental causes (Ref. 171). Most studies have found no significant association between RAGE expression and IUGR pathophysiology; however, Alexander et al. reported decreased RAGE protein concentrations in placental tissue from mothers of IUGR-affected infants (Ref. 116) (Table 8).

In other neonatal conditions, upregulation of RAGE and its ligands has been consistently observed across multiple tissues. Regers et al. conducted a comprehensive analysis of circulating sRAGE and S100B levels in plasma from extremely preterm infants and demonstrated that higher sRAGE concentrations were inversely correlated with both sepsis incidence and FiO₂ (fraction of inspired oxygen) requirements, suggesting a protective role against early respiratory morbidity (Ref. 172). Similarly, Lu et al. reported that alterations in HMGB1 and sRAGE levels in cord blood were directly linked to the degree of systemic inflammation and correlated with the severity of brain injury, particularly among infants with severe neurological impairment. The authors proposed that combined monitoring of HMGB1 and sRAGE levels could serve as a prognostic biomarker for predicting brain injury in preterm neonates (Ref. 173).

In contrast, Germanova et al. found no association between RAGE polymorphisms and intrahepatic cholestasis of pregnancy (Ref. 122). Conversely, Zhao et al. demonstrated increased concentrations of RAGE ligands, S100A8, S100A9 and S100A12, in both neonatal peripheral blood and placental tissue in cases of intrauterine hepatitis B virus (HBV) infection, a condition strongly linked to hepatic dysfunction (Ref. 174).

Collectively, current evidence indicates that RAGE and its ligands exert context-dependent effects in neonatal and infant morbidities. While RAGE downregulation may impair placental signalling and foetal growth in IUGR, excessive activation of the RAGE axis and elevated ligand levels appear to exacerbate inflammatory and oxidative stress-related complications in preterm or infected infants. Further integrative studies combining molecular, genetic and clinical data are warranted to elucidate the precise contribution of RAGE signalling to neonatal health and disease.

### Other benign gynaecological conditions, systemic diseases with gynaecological implications and physiological age-related changes

The role of RAGE has also been explored in several benign gynaecological conditions, although to a lesser extent than in the previously discussed disorders. The available evidence, summarized in Table 9, reveals that alterations in RAGE signalling are not disease-specific but rather reflect broader dysregulation of inflammatory and metabolic pathways that underlie many gynaecological disorders.

Antiphospholipid syndrome (APS) represents a systemic autoimmune condition characterized by thromboembolic events and recurrent pregnancy complications. Both studies included in Table 9 demonstrated increased concentrations of HMGB1 and its soluble receptor sRAGE in the serum of APS patients (Refs 175, 176). This likely represents an inflammatory response in which HMGB1 acts as a prothrombotic mediator promoting platelet activation, while sRAGE serves a compensatory, anti-inflammatory decoy. The simultaneous upregulation of both molecules suggests that RAGE-associated mechanisms may contribute to the endothelial dysfunction and hypercoagulable state typical of APS and that their combined measurement could serve as a prognostic indicator of thrombotic risk.

In pelvic organ prolapse (POP), a common gynaecological condition, affecting approximately 40% of women aged 45–85 years, three independent studies consistently demonstrated increased expression of AGEs and RAGE in vaginal tissue samples (Refs 177, 178, 179). These findings support the concept that AGE–RAGE signalling disrupts extracellular matrix (ECM) homeostasis by enhancing MMP-1 expression and reducing type I collagen synthesis. Such molecular remodelling weakens connective tissue architecture, ultimately predisposing to organ descent. Experimental inhibition of the AGE–RAGE axis restored normal collagen balance in fibroblast cultures, underscoring its direct mechanistic involvement in POP pathophysiology.

Cervical insufficiency, the inability of the cervix to maintain a pregnancy in the absence of uterine contractions and a leading cause of spontaneous preterm birth, also appears to involve dysregulated RAGE signalling. Elevated concentrations of RAGE and its ligands EN-RAGE (S100A12) and S100A8/A9 in amniotic fluid (Refs 180, 181) indicate activation of inflammatory pathways at the maternal–foetal interface. These proteins are known to amplify NF-κB–dependent cytokine release and leukocyte recruitment, suggesting that aberrant RAGE activation may contribute to premature cervical remodelling. This observation parallels findings in pregnancy-associated disorders such as preeclampsia and recurrent miscarriage, where RAGE–ligand upregulation likewise drives inflammation-driven tissue damage.

Ageing-related reproductive decline provides another context in which RAGE signalling has been explored. Across multiple studies, plasma and follicular fluid levels of sRAGE decreased with advancing maternal age (Refs 182, 183, 184). Given the decoy function of sRAGE, this decline likely reflects reduced anti-inflammatory buffering capacity and greater vulnerability to AGE accumulation. The positive association between follicular sRAGE levels and ovarian reserve parameters (e.g., oocyte count and quality) further supports this interpretation. Stensen et al. (Ref. 185) demonstrated age-dependent increases in AGE-binding capacity in granulosa–lutein cells, indicating cumulative glycation stress within the ovarian microenvironment. Intriguingly, one study (Ref. 186) reported that lower sRAGE levels were linked to higher embryo quality during in vitro fertilization (IVF), suggesting that reduced sRAGE may also signal a lower AGE burden under controlled conditions, highlighting the context-dependent nature of RAGE signalling in fertility.

Finally, recent investigations have extended RAGE research into infection-related and metabolic settings. Elevated serum HMGB1 with concomitant reduction in sRAGE was observed in patients with diverse benign gynaecological diseases (Ref. 27), suggesting that systemic inflammation suppresses the soluble receptor’s protective role. Similarly, COVID-19–related studies identified increased concentrations of methylglyoxal and glycated albumin (AGEs) in pregnant women with SARS-CoV-2 infection (Ref. 187), supporting the hypothesis that infection-induced oxidative stress enhances AGE–RAGE pathway activation.

Collectively, data summarized in Table 9 indicate that RAGE and its ligands participate in a shared inflammatory–metabolic axis influencing a wide range of gynaecology-related states. While the direction of change (increase or decrease) varies by disease and tissue type, the consistent involvement of the RAGE pathway underscores its potential as both a biomarker and a therapeutic target. Further mechanistic and longitudinal studies are required to determine whether modulation of RAGE signalling could mitigate tissue remodelling, inflammation and age-related reproductive decline.

## The presence of RAGE in gynaecological malignancies

Cancer remains one of the most significant global public health challenges. According to data from the American Cancer Society, over two million new cancer cases and more than six hundred thousand cancer-related deaths are expected to occur in 2025, making cancer the second leading cause of mortality in the United States and the primary cause of death among individuals under 85 years of age (Ref. 188). By 2040, global incidence is projected to reach 28 million new cases and 16.2 million deaths. Because the development of targeted anticancer therapies remains one of the most effective long-term strategies, continued identification and characterization of novel molecular pathways driving tumourigenesis is essential (Ref. 189).

In recent years, growing evidence has implicated RAGE in the pathogenesis of multiple malignancies. Elevated *RAGE* expression has been described in gastric, hepatic, endometrial and oral squamous cell carcinomas. Interestingly, lung cancer demonstrates an opposing pattern, with reduced *RAGE* expression but increased ligand levels. Functional studies indicate that RAGE–ligand interactions contribute to tumour progression by promoting proliferation, enhancing autophagy, inhibiting apoptosis and increasing cellular invasiveness and metastatic potential (Ref. 19). Conversely, pharmacological inhibition of RAGE, for example, with the small-molecule antagonist such as FPS-ZM1, has been shown to suppress cancer cell viability and induce apoptosis in vitro (Ref. 190).

Among gynaecological malignancies, cervical cancer is one of the most extensively studied in relation to RAGE signalling. As summarized in Table 10, increased expression of RAGE and its ligands HMGB1, S100A7 and S100A9 have been consistently observed in malignant cervical tissue (Refs 191, 192, 193, 194). Immunohistochemical analyses revealed a stepwise elevation in RAGE and S100A9 expression from chronic cervicitis through cervical intraepithelial neoplasia to invasive squamous carcinoma, suggesting their involvement in the neoplastic transformation process (Ref. 194). Interestingly, RAGE expression was highest in well-differentiated squamous carcinomas compared with poorly differentiated ones (Ref. 194). Circulating sRAGE levels, in contrast, are significantly reduced in the peripheral blood of cervical cancer patients (Ref. 195), reflecting either receptor sequestration or increased ligand binding. Genetic analyses identified the 82G>S single nucleotide polymorphism (SNP) in the AGER gene as a potential susceptibility factor for cervical cancer (Ref. 195). Parallel findings were reported by Zhang et al., who observed the same polymorphism in ovarian cancer cohorts (Ref. 196). Together, these studies suggest that both transcriptional and genetic mechanisms underlie RAGE dysregulation in cervical tumourigenesis.

Similar patterns were identified in ovarian carcinoma, where RAGE and its ligands are markedly upregulated at both the tissue and peripheral blood (Refs 27, 197, 198). Elevated HMGB1 levels in patient serum, coupled with decreased concentrations of sRAGE, reflect a shift towards proinflammatory RAGE activation (Ref. 27). Bioinformatic and experimental studies revealed that the RAGE ligand S100B is overexpressed in ovarian cancer tissues and correlates with advanced tumour stage, poor differentiation and reduced survival (Ref. 198). Knockdown of S100B suppressed self-renewal and tumourigenicity, indicating that RAGE–S100B signalling may promote ovarian cancer aggressiveness. Interestingly, data from the ROLANDO–GEICO clinical trial suggested that in patients with platinum-resistant ovarian cancer, higher circulating RAGE levels were associated with lower progression risk and improved survival (Ref. 199). This apparent discrepancy may reflect distinct roles for membrane-bound versus soluble RAGE isoforms, emphasizing the need for isoform-specific investigation in clinical settings.

In endometrial cancer, multiple studies confirm significant *RAGE* overexpression in tumour tissue, particularly in poorly differentiated adenocarcinomas (Refs 200, 201, 202). Given that nearly 40% of endometrial cancer cases are associated with metabolic disorders such as obesity and diabetes, dysregulated metabolic signalling is thought to be a major driver of tumour initiation and progression. RAGE expression positively correlates with microvessel density and tumour volume, consistent with its role in angiogenesis and metabolic reprogramming. In xenograft mouse models, RAGE knockdown reduced microvessel formation and tumour growth (Ref. 200). Importantly, anti-RAGE antibody–drug conjugates (ADCs) selectively eliminated endometrial cancer cells in vitro and demonstrated safety in murine models, providing a rationale for the therapeutic targeting of RAGE in endometrial malignancies (Ref. 202).

While RAGE itself acts predominantly as a pro-tumourigenic factor in endometrial cancer, the behaviour of its ligands appears to be more complex and context-dependent. Among them, HMGB1 has emerged as a particularly intriguing molecule with dual functional roles. Although HMGB1 promotes invasion and inhibits apoptosis in malignancies such as melanoma and cutaneous squamous cell carcinoma, its expression is reduced in endometrial cancer tissues. Functional assays by Luan et al. demonstrated that HMGB1 knockdown enhanced invasion and metastasis, likely through induction of epithelial–mesenchymal transition (EMT) (Ref. 203). These findings suggest a potential tumour-suppressive role of HMGB1 in specific gynaecological contexts, underscoring the context-dependent nature of RAGE–ligand signalling and the importance of microenvironmental factors in modulating its downstream effects.

Although not a female reproductive system malignancy per se, breast cancer provides additional evidence for the oncogenic relevance of RAGE ligands. Elevated expression of S100A8 in both tumour cells and adjacent stromal tissue is strongly associated with poor survival across molecular subtypes, independent of hormone receptor status (Ref. 204). This highlights the potential of RAGE-associated ligands as prognostic biomarkers beyond classical receptor-based classifications.

As summarized in Table 10, most studies demonstrate a global upregulation of RAGE and its ligands (particularly HMGB1 and S100 family proteins) across gynaecological malignancies, accompanied by reduced circulating levels of sRAGE. This imbalance reflects sustained RAGE pathway activation and enhanced ligand engagement within the tumour microenvironment. The AGE–RAGE axis likely amplifies oxidative stress, inflammation and angiogenesis, establishing a self-perpetuating loop that promotes tumour growth, invasiveness and immune evasion. Nevertheless, notable discrepancies, such as the inverse prognostic association of RAGE expression in platinum-resistant ovarian cancer or the tumour-suppressive effect of HMGB1 in endometrial carcinoma, underscore the context-dependent nature of RAGE signalling, a recurring theme in current research. These variations may arise from differences in tumour histotype, ligand repertoire, receptor isoform distribution or metabolic state. Collectively, current evidence supports the concept that RAGE acts as a pleiotropic regulator of tumour biology, influencing proliferation, apoptosis, invasion and angiogenesis in a context-specific manner. Its broad expression profile and capacity to engage multiple ligands make it an appealing, yet mechanistically complex, therapeutic target. Future research should prioritize elucidating isoform-specific functions (membrane-bound vs. soluble RAGE) and identifying predictive biomarkers to optimize the design of RAGE-targeted therapies in gynaecologic oncology.

## RAGE as a precision biomedicine therapeutic target for gynaecological diseases

### Animal models in the study of RAGE-mediated gynaecological and pregnancy-related disorders

Although analyses of patient-derived tissues remain the most reliable source of information on RAGE-mediated interactions in gynaecological diseases, the need for advanced functional studies has led to the development of animal models that closely mimic pathologically altered states. Pharmacologically induced models, while not fully replicating the natural course of disease, provide a reasonable approximation for most experimental purposes and are ethically acceptable for investigating embryopathies and developmental defects. Over the years, several animal models have been established to study RAGE-mediated mechanisms, most commonly using mice or rats exposed to chemical agents that induce metabolic or inflammatory states. Table 11 summarizes up-to-date studies analysing RAGE expression in animal models of gynaecological and pregnancy-associated disorders.

Analysis of these studies reveals that alterations in RAGE expression and signalling patterns largely mirror those observed in human tissues. Increased RAGE and AGE levels are consistently reported in models of gestational diabetes mellitus, preeclampsia and polycystic ovary syndrome, supporting their relevance for investigating RAGE-mediated inflammation and metabolic dysregulation. Similarly, changes in RAGE and HMGB1 expression observed in intra-amniotic infection and developmental abnormalities (such as cleft palate or intrauterine growth restriction) highlight the receptor’s involvement in foetal development and maternal–foetal immune communication.

Collectively, these findings indicate that animal models represent a valuable tool for exploring the mechanistic role of RAGE in gynaecological pathology. Nonetheless, further studies integrating molecular, histological and functional assessments are needed to fully elucidate how RAGE contributes to disease onset and progression.

### Targeting RAGE signalling in gynaecology

#### RAGE modulation as a potential therapeutic strategy

**Figure 4.**
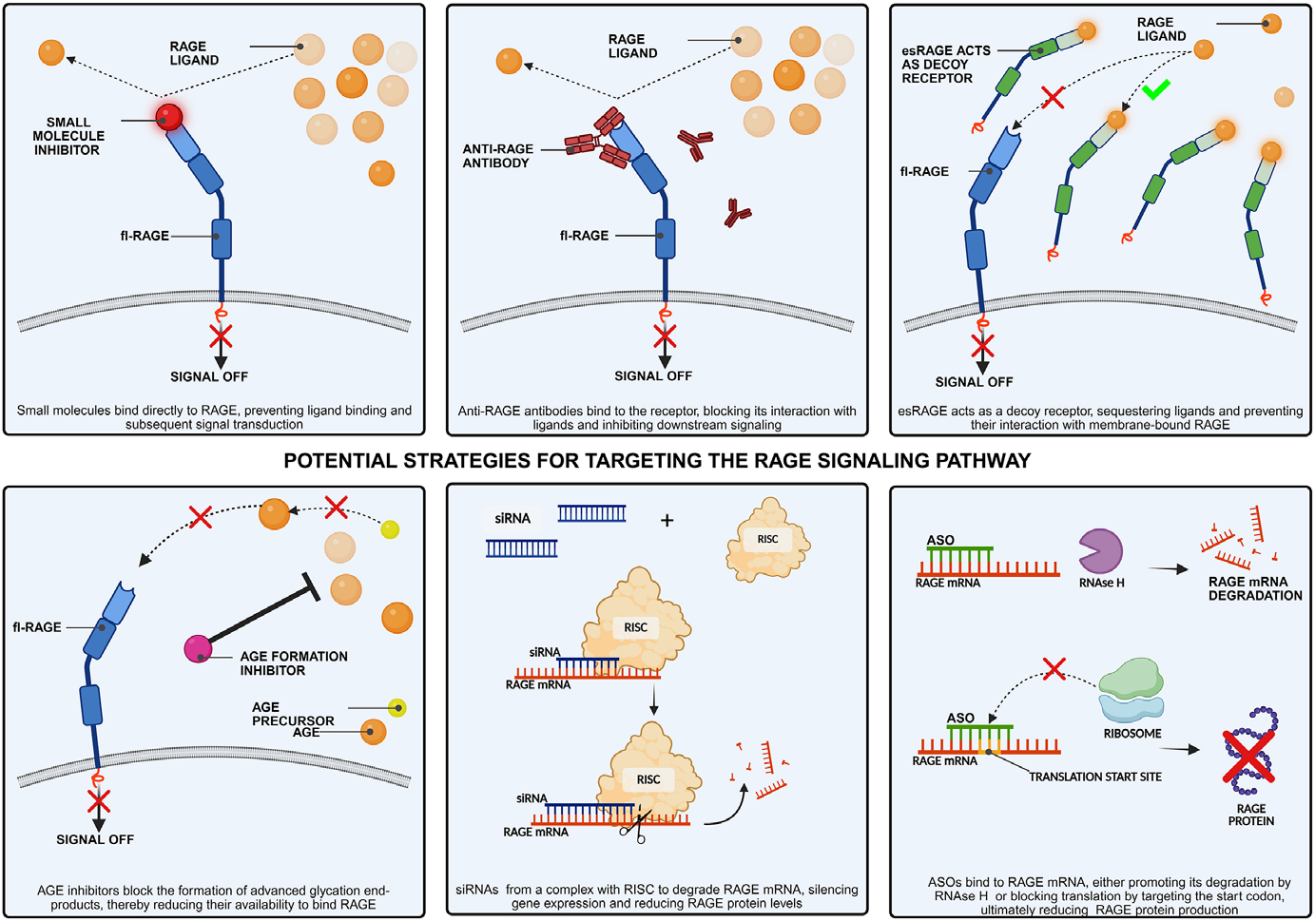
Inhibition of the RAGE pathway at different target points. Top row: RAGE signalling can be inhibited by small molecule antagonists, neutralizing antibodies, or soluble decoy receptors such as esRAGE, which sequester ligands and prevent receptor activation. Bottom row: Additional strategies include inhibition of AGE formation, as well as suppression of RAGE expression through siRNA-mediated mRNA degradation or antisense oligonucleotides (ASOs) that block translation or induce RNA cleavage. Abbreviations: AGE: advanced glycation end-products; ASO: antisense oligonucleotide; esRAGE: endogenous secretory receptor for advanced glycation end-products; fl-RAGE: full-length receptor for advanced glycation end-products; mRNA: messenger ribonucleic acid; RISC: RNA-induced silencing complex; RNAse H: ribonuclease H; siRNA: small-interfering ribonucleic acid. Created in BioRender. Łuszczyński, K. (2026) https://BioRender.com/syxg8v4. Figure 4. long description.

Given the central role of RAGE in inflammatory signalling, modulation of this pathway has emerged as a promising therapeutic concept. Various strategies have been developed to inhibit RAGE at different points of its signalling cascade, ranging from direct receptor blockade to interference with ligand availability and suppression of RAGE expression (Figure 4). Additionally, several clinically used drugs also exhibit RAGE-modulating properties in addition to their primary mechanisms of action. In this section, we summarize current evidence and experimental approaches exploring RAGE modulation as a potential therapeutic strategy.

The first category of RAGE inhibitors includes molecules that directly block the ligand-binding domain of the receptor, thereby preventing activation by pro-inflammatory ligands. The most promising results have been achieved with small-molecule inhibitors such as FPS-ZM1 and TPP488 (azeliragon), which directly disrupt ligand–receptor interactions and have already undergone clinical testing. Monoclonal antibodies targeting RAGE have also shown efficacy *in vitro* and in animal models, significantly reducing RAGE-mediated signalling, although they have not yet advanced to clinical trials (Ref. 202).

A second strategy involves interfering with RAGE ligands, such as AGEs, HMGB1 and S100 proteins, to limit receptor activation. For this purpose, recombinant soluble RAGE proteins (sRAGE), acting as natural decoy receptors, sequester circulating ligands and prevent their binding to membrane-bound RAGE. In a study by Jeong et al., mice treated with an sRAGE-Fc fusion protein exhibited reduced expression of pro-inflammatory cytokines (IL-1β, TNFα, IL-6) and improved survival in sepsis (Ref. 225). Similarly, small molecules that inhibit AGE formation, such as aminoguanidine or pyridoxamine (a natural form of vitamin B6), were shown to suppress toxic AGE (tAGE)-mediated β-tubulin aggregation, promote neurite outgrowth and reduce tau phosphorylation, thereby attenuating diabetes-associated Alzheimer’s disease pathology (Ref. 226).

Genetic approaches have also been explored to suppress *RAGE* expression. siRNA-based silencing and antisense oligonucleotides (ASOs) both demonstrated efficacy in preclinical models. For example, Cai et al. reported that *RAGE*-targeted siRNA reduced *RAGE* mRNA and protein levels in rat liver fibrosis, correlating with decreased inflammation and fibrosis progression (Ref. 227). Likewise, Kuniyasu et al. showed that treatment with antisense S-oligodeoxynucleotides reduced *RAGE* expression and invasive potential in MKN28 gastric cancer cells (Ref. 228).

Beyond receptor- and ligand-directed therapies, several non-specific RAGE inhibitors have been identified. This group includes natural phytochemicals – such as polyphenols, organic acids and flavonoids – which modulate the RAGE axis while simultaneously lowering reactive oxygen species (ROS) levels (Ref. 229). In addition, clinically used drugs such as metformin (Ref. 230), statins (Ref. 231) and angiotensin II receptor blockers (sartans) (Ref. 232) have demonstrated RAGE-inhibitory activity.

Because of the complex structure and pleiotropic functions of RAGE, its inhibition may impact multiple cell types and biological processes. To date, clinical trials have primarily tested RAGE inhibitors in neurological disorders, such as Alzheimer’s disease (Ref. 233), and in oncology, including glioblastoma (Ref. 234) and breast cancer (Ref. 235). While these studies confirmed the safety of azeliragon, the limited number of trials prevents clear conclusions about therapeutic efficacy. In gynaecology, further research is required to validate the clinical potential of RAGE-modulating therapies. Localized delivery, for example, via intrauterine devices (IUDs) or vaginal formulations, could provide targeted inhibition while minimizing systemic side effects. However, additional clinical studies are necessary to fully establish the therapeutic relevance of RAGE modulation in gynaecological disorders.

#### Current challenges in implementation of RAGE-targeting therapies for clinical settings

Although selective targeting of the receptor for advanced glycation end-products could a potent therapeutic strategy, there are still several important questions that should be answered before implementation.

First of all even though RAGE function is mainly linked with pathologic overactivation during chronic inflammation, RAGE plays also physiological role in normal cellular function raising questions about the long-term effects of the complete blockade (Ref. 49). Gathered data indeed showed that infection in RAGE deficient mice resulted in prolonged healing wound healing and compromised immune response (Ref. 236). Moreover, as RAGE functions as a phosphatidylserine receptor and assists in the clearance of apoptotic cells, complete RAGE silencing could lead to accumulation of apoptotic cells and cellular debris (Ref. 54). Additionally, as showed by Li et al. in a rat model of subarachnoid haemorrhage (SAH), pharmacological inhibition of RAGE resulted in reduction of neuroinflammation, brain oedema and improved neurological function at day 1. However, RAGE inhibition also increased neuronal apoptosis, reduced autophagy and cell death, suggesting a protective effect of RAGE activation against SAH-induced neuronal damage. This dual role of RAGE highlights the need for caution in RAGE-targeted therapies (Ref. 237). Additionally, given the high level of natural RAGE expression in lungs, complete down-regulation of RAGE could negatively impact lung homeostasis. Therefore, RAGE inhibition should be proceeded with caution basing on results from pre-clinical and the earliest stages of clinical trials.

Second, because RAGE is a complex molecule with many ligands other than AGEs, inhibiting just one ligand may not be enough to stop the course of inflammation and tissue degradation. Additionally, physiological obstacles such as the blood–brain barrier or the blood–eye barrier may restrict inhibitors’ absorption, reducing their therapeutic potential. These intricate relationships may impede the straightforward application of preclinical results to clinical practice. As demonstrated in the Phase III STEADFAST research examining the effectiveness of Azeliragon in treating Alzheimer’s disease, individuals using the medicine did not exhibit substantial cognitive gains; moreover, greater inhibitor dosages were linked with confusion and increased fall risk. On the other hand, past Phase II trial results indicated that a smaller 5-mg dosage might have favourable benefits (Ref. 238). Aminoguanidine was a non-selective RAGE inhibitor evaluated in a Phase III ACTION II trial to test its potential to slow the progression of renal failure in diabetes mellitus type II patients; however, due to inhibitor toxicity paired with high doses needed for clinical effects, drug is no longer used in clinical practice (Ref. 239). On the other hand, Pyridoxamine, another non-selective RAGE inhibitor tested in a PIONEER-CSG-17 study, proved effective in slowing the progression of renal failure in diabetic nephropathy (Ref. 240). Therefore, results acquired during pre-clinical trials should be implemented with caution into clinical settings.

Finally, effective and targeted delivery of RAGE inhibitors to the disease site is critical for limiting potential side effects. Although no such clinical trials have yet been conducted, preclinical studies have shown that anti-RAGE antibody-drug conjugates preferentially eliminate endometrial cancer cells in vitro and are safe in animal models (Ref. 202). As a result, further research is needed to turn such pathways into functional medications.

As previously stated, actual implementation of RAGE inhibitors into clinical practice is a challenging problem due to several anatomical limitations and physiological processes. However, given the encouraging outcomes of numerous RAGE inhibitors in preclinical trials, further study is needed to fully grasp their therapeutic potential and potential limitations.

#### RAGE-modulating therapeutics in gynaecology: Current evidence and efficacy

Growing evidence suggests that RAGE plays a critical role in the pathophysiology of several gynaecological conditions. This has prompted investigations into the therapeutic potential of RAGE inhibition. Table 12 provides a summary of preclinical and bioinformatic studies exploring the modulatory effects of various compounds on RAGE signalling across a range of gynaecological and pregnancy-related pathologies. The most frequently studied RAGE modulators are naturally occurring compounds derived from traditional herbal medicines, particularly those used in traditional Chinese medicine (TCM). Several studies also assessed the impact of known pharmaceuticals (e.g., metformin), vitamins (e.g., vitamin D3), antioxidant agents (e.g., alpha-lipoic acid, indole-3-propionic acid) and recombinant proteins, as well as synthetic RAGE inhibitors such as Azeliragon and FPS-ZM1. Additionally, genetic interventions, including siRNA-mediated RAGE knockdown or knockout, have been evaluated in selected models.

In one of the most extensively studied conditions, that is, PCOS, several bioinformatic analyses identified herbal formulations, such as Cangfu Daotan Decoction, Leonuri Herba and Erxian Decoction, as potential non-specific RAGE inhibitors that downregulate AGE–RAGE signalling. While promising, these findings require validation in experimental models.

Among tested agents, L-carnitine demonstrated substantial efficacy in PCOS mouse models, reducing oxidative stress, restoring estrous cyclicity, improving oocyte quality and reducing hormonal imbalance through suppression of RAGE and AGE expression (Refs 213, 214). Furthermore, PEDF (pigment epithelium-derived factor), a multifunctional secreted protein with anti-inflammatory and anti-angiogenic activity, was shown to downregulate AGE-mediated secretion of pro-inflammatory and pro-angiogenic cytokines in PCOS (Ref. 212).

RAGE-modulating therapies have also been evaluated in models of ovarian ageing and failure. Treatment with herbal formulations such as Zishen Yutai Pill or Erxian Decoction significantly improved ovarian reserve parameters in chemotherapy-induced primary ovarian insufficiency models (Refs 241, 242). Additionally, antioxidant agents including vitamin D3, vitamin B6 (pyridoxamine), alpha-lipoic acid and indole-3-propionic acid have shown ovary-protective effects by downregulating RAGE expression, reducing oxidative damage and AGEs-induced cellular damage, as well as restoring hormonal balance (Refs 243, 244, 245, 246).

In endometriosis models, RAGE inhibition using natural flavonoids such as neferine and Guizhi Fuling Wan resulted in reduced fibrosis, decreased cell invasiveness and increased apoptosis in ectopic endometrial cells (Refs 247, 248).

In pelvic inflammatory disease and cervical inflammation, herbal and synthetic inhibitors significantly reduced inflammatory cytokine levels and local immune cell activation (Refs 205, 249). Silencing of RAGE using siRNA in fibroblasts derived from patients with pelvic organ prolapse led to reduced matrix degradation and increased collagen type I synthesis, suggesting its potential therapeutic utility (Ref. 178).

RAGE-targeted strategies have been investigated in pregnancy-related conditions. In gestational diabetes mellitus (GDM), pharmacological agents (metformin, baicalin, crocetin, betaine, sRAGE) and genetic interventions (e.g., RAGE knockout) improved placental function by reducing oxidative stress, inflammation and permeability, thereby limiting foetal abnormalities (Refs 208, 220, 224, 250, 251). These interventions were also associated with increased foetal birth weight and a lower risk of developmental defects. Comparable benefits have been observed in preeclampsia (PE) and unexplained recurrent spontaneous abortion (URSA). In PE, bioinformatic analysis has shown that resveratrol inhibited RAGE and downstream inflammatory mediators, thereby potentially slowing disease progression (Ref. 252). In a murine model of URSA, aspirin acted as an HMGB1 inhibitor and conferred protection by suppressing pyroptosis and preventing maternal–foetal interface damage (Ref. 169).

In gynaecological malignancies, RAGE inhibition was associated with reduced tumour growth and invasiveness. In endometrial cancer models, interventions included resveratrol, timosaponin A3, antibody–drug conjugates targeting RAGE and siRNA knockdown, all of which consistently decreased tumour volume, angiogenesis and hormone dysregulation (Refs 200, 202, 253). Interestingly, RAGE-targeting therapies were highly specific and had little effect on healthy tissue (Ref. 202). In ovarian cancer, plant-based modulators such as Acacetin or Sophorae Flavescentis Radix, as well as knockdown of RAGE ligands (e.g., S100B), reduced cancer cell viability and suppressed tumour stemness (Refs 198, 254, 255). Similar therapeutic responses were observed in cervical and breast cancer, where RAGE inhibition led to decreased cellular proliferation, migration and increased apoptosis (Refs 256, 257, 258).

Taken together, these findings provide compelling evidence that RAGE inhibition – either through natural compounds, pharmaceuticals or genetic tools – holds significant therapeutic potential in both benign and malignant gynaecological conditions, as well as in pregnancy-related disorders. Nevertheless, the majority of current data is derived from preclinical models and requires validation in clinical settings.

## The role of RAGE and its ligands as potential clinical biomarkers

The potential of RAGE and its ligands as clinically relevant biomarkers has already been explored in several medical specialties. Evidence from these fields suggests that distinct RAGE isoforms and ligands may provide diagnostic, prognostic or risk-stratification value, although their utility appears to be strongly disease-specific.

Cardiology is one of the most rapidly developing areas of medicine in terms of novel biomarker implementation, and several studies have suggested that RAGE-related molecules may have clinical utility in cardiovascular disease. In pulmonary arterial hypertension (PAH), a progressive disorder with a strong need for minimally invasive diagnostic and monitoring strategies, Diekmann et al. demonstrated that plasma sRAGE concentrations were significantly elevated and outperformed the conventional heart failure biomarker N-terminal pro-brain natriuretic peptide (NT-proBNP) (Ref. 267). In coronary artery disease (CAD), where inflammation plays a central role in disease development and progression, Wang et al. showed that circulating sRAGE and S100A12 levels were significantly increased in patients with acute coronary syndrome (Ref. 268). In contrast, Larsen et al. reported that persistently high sRAGE levels after hospital discharge were associated with a significantly increased risk of recurrent major cardiovascular events, worsening left ventricular function, and the development of heart failure (Ref. 269). Similarly, Reichert et al. found that elevated sRAGE concentrations were associated with higher cardiovascular risk in patients with established CAD (Ref. 270). Collectively, these findings suggest that sRAGE may serve as a clinically useful biomarker for risk stratification in selected cardiovascular conditions. In hypertrophic obstructive cardiomyopathy (HOCM), Li et al. demonstrated an opposite pattern, showing that lower circulating sRAGE levels were associated with a higher incidence of severe cardiovascular events and greater myocardial fibrosis (Ref. 271).

In pulmonology, where chronic inflammation underlies numerous disorders such as asthma and chronic obstructive pulmonary disease (COPD), RAGE-mediated signalling has also been extensively investigated for its diagnostic and prognostic value. In a multicentre study, Pratte et al. showed that lower plasma or serum sRAGE concentrations were significantly associated with a diagnosis of COPD, reduced FEV1 and greater emphysema severity (Ref. 272). Similar observations were reported by Kamel et al., who demonstrated a strong correlation between decreased sRAGE levels and COPD severity (Ref. 273), and by Iwamoto et al., who identified reduced sRAGE concentration as a predictor of progressive decline in lung function assessed by spirometry (Ref. 274). In asthma, higher circulating sRAGE levels have been associated with lower systemic inflammation and better pulmonary function (Ref. 275), whereas significantly reduced sRAGE concentrations were observed in patients with poorly controlled disease (Ref. 276). In addition, increased concentrations of RAGE ligands such as HMGB1 (Refs 277, 278, 279) and S100A9 (Ref. 280) in airway secretions were linked to asthma severity and disease control. Similar observations have been made in idiopathic pulmonary fibrosis (IPF), where reduced plasma sRAGE levels were proposed as a marker of disease severity (Ref. 281), while Kitadai et al. showed that low sRAGE levels combined with selected RAGE polymorphisms may help identify patients at increased risk of acute exacerbation (Ref. 282).

Oncology represents another field in which attempts have been made to implement RAGE-related molecules as biomarkers of disease course and prognosis. In clear cell renal cell carcinoma, RAGE overexpression was associated with shorter metastasis-free and overall survival (Ref. 283). Likewise, in hepatocellular carcinoma, Ito et al. reported that increased RAGE expression correlated with poor therapeutic outcome (Ref. 284). However, the opposite pattern has been described in lung cancer, where loss of the physiologically high RAGE expression characteristic of healthy lung tissue was associated with poor prognosis and impaired immune cell infiltration (Refs 285, 286). In addition to tissue expression, soluble RAGE may also have predictive value in selected malignancies. For example, White et al. demonstrated that low circulating sRAGE levels in post-menopausal women were associated with an increased risk of pancreatic cancer (Ref. 287).

To date, gynaecological research indicates a general trend in which increased expression of full-length RAGE and its activating ligands, together with downregulation of inhibitory isoforms such as sRAGE and esRAGE, correlates with disease incidence and severity. However, no studies have yet established quantitative diagnostic thresholds for specific RAGE isoforms or their ligands in gynaecological disorders. Furthermore, as observed in other fields of medicine, changes in the expression or concentration of RAGE ligands and isoforms may yield divergent, and sometimes even contradictory, results depending on the disease context. This underscores the need to interpret RAGE-related alterations in a disease-specific manner. Although RAGE isoform diversity arises from both alternative splicing and post-translational modifications, the disease-specific role of these alterations in gynaecological disorders remains poorly understood. This represents an important gap in the current literature, particularly in relation to ligand affinity, receptor trafficking and cell type-specific signalling. Therefore, further studies are needed to fully define the biomarker potential of RAGE-related molecules.

## Conclusions

**Figure 5.**
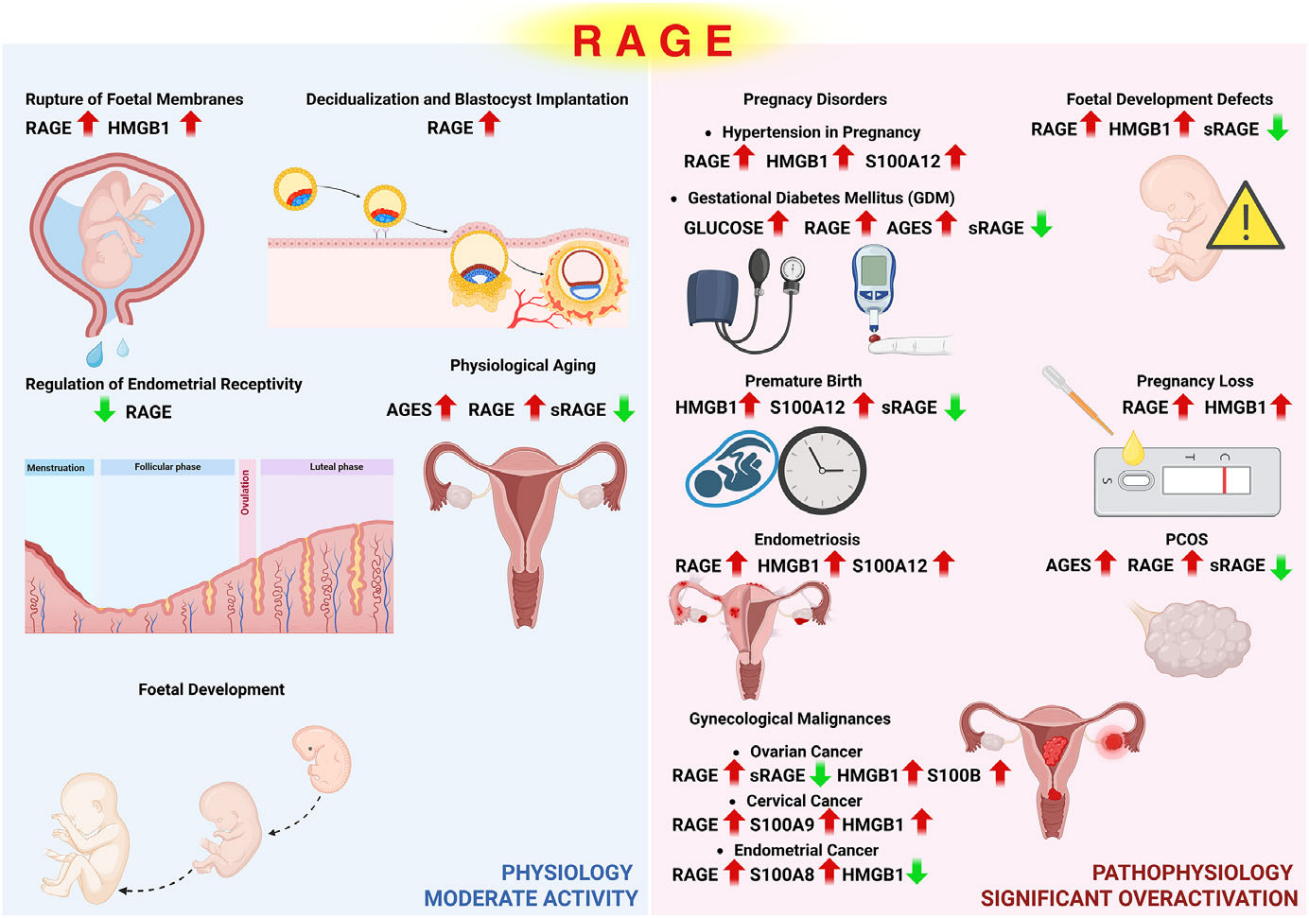
The comparison of RAGE-mediated signalling in physiological and pathological conditions in female reproductive system. During physiological processes, a mild activation of RAGE occurs. On the other hand, during pathological states, a significant overactivation of RAGE occurs. Abbreviations: AGE: advanced glycation end-product; EN-RAGE: extracellular newly identified receptor for advanced glycation end-products binding protein; HMGB1: high mobility group box 1 protein; RAGE: receptor for advanced glycation end-products; sRAGE: soluble receptor for advanced glycation end-products. Figure 5. long description.

RAGE-mediated signalling pathways play a pivotal role in both the physiology and pathology of gynaecological tissues. Accumulating evidence indicates that RAGE acts as a central molecular hub integrating metabolic, inflammatory and angiogenic signals across a broad spectrum of gynaecological disorders, from benign conditions and pregnancy-related complications to malignant neoplasms. In most cases, aberrant RAGE activation, coupled with elevated ligand concentrations and reduced levels of soluble decoy receptors, sustains chronic inflammation and oxidative stress, thereby establishing a self-perpetuating loop that drives tissue damage and disease progression (Figure 5).

Distinct RAGE isoforms exhibit diverse biological functions, acting either as signalling receptors (membrane-bound forms) or as regulatory decoys (soluble forms). Despite the growing recognition of their importance, few studies adequately differentiate between these isoforms, which significantly hampers the development of isoform-specific diagnostic markers and therapeutic strategies. Likewise, the absence of robust experimental models that faithfully reproduce RAGE–ligand interactions in reproductive tissues remains a major obstacle to translational progress.

Emerging preclinical evidence demonstrates that pharmacological or genetic inhibition of RAGE can mitigate inflammation, fibrosis, and tumourigenesis in multiple gynaecological contexts, underscoring the receptor’s potential as a therapeutic target. However, translating these findings into clinical practice will require comprehensive, multidisciplinary approaches integrating molecular biology, omics technologies and advanced disease modelling. Future research should focus on elucidating isoform-specific signalling mechanisms, identifying predictive biomarkers of RAGE pathway activation and optimizing the design of targeted modulators, potentially in combination with existing hormonal or metabolic therapies.

Taken together, current evidence underscores the central role of RAGE in gynaecological pathophysiology, but its full therapeutic potential has yet to be realized. Continued investigation at the intersection of molecular pathology, pharmacology and reproductive medicine will be crucial to bridge the gap between bench and bedside and to establish RAGE as a viable target in precision gynaecologic care.

## Data Availability

All relevant data are included.

## Long descriptions

### Figure 1. Long description

The flowchart is organized into two main columns under the headers Identification of studies via databases and registers and Identification of studies via other methods.

Left Column: Identification of studies via databases and registers.

- Identification stage: Records identified from Databases (n = 833). An arrow points to a box for Records removed before screening, which includes Duplicate records removed (n = 423) and Records written in language different than English (n = 17).

- Screening stage: Records screened (n = 393) leads to Records excluded (n = 212). Below this, Reports sought for retrieval (n = 181) leads to Reports not retrieved (n = 1). Then, Reports assessed for eligibility (n = 180) leads to a large box of Reports excluded, containing: Research papers not related to the field of gynecology (n = 12), Research papers not related to the topic of R A G E (n = 12), Review articles and meta-analysis (n = 12), and Papers retracted by the publisher (n = 1).

- Full-Text Analysis stage: Reports analysed in detail (n = 143).

Right Column: Identification of studies via other methods.

- Identification stage: Records identified from Manual Citation Searching (n = 10).

- Screening stage: Reports sought for retrieval (n = 10) leads to Reports not retrieved (n = 0). Reports assessed for eligibility (n = 10) leads to Reports excluded (n = 0).

Final Stage: Arrows from both the Reports analysed in detail (left) and Reports assessed for eligibility (right) converge at the bottom box: New studies included in review (n = 153).

Navigate back to Figure 1..

### Table 1. Long description

The table consists of eight columns: Year, Publication, State, Sample size, Control size, Material, Analysis method, and Marker.

* 2004, Reference 75: Healthy pregnancy. Sample and control sizes are N/A. Material: Chorionic villi. Methods: I H C and Western blot. Marker: R A G E is present.

* 2003, Reference 76: Healthy pregnancy. Sample size 10, Control size 10 non-pregnant. Material: Myometrium and omental fat. Method: I H C. Marker: increased R A G E.

* 2020, Reference 77: Healthy pregnancy. Sample: Foetal membrane zone of altered morphology. Control: Foetal membrane zone of intact morphology. Material: Foetal membrane. Methods: I F, q R T-q P C R, and Western blot. Markers: increased H M G B 1 and increased R A G E in choriodecidua; significantly increased H M G B 1 and R A G E in amnion.

* 2023, Reference 78: Two states. 1. Healthy pregnancy: Sample and control N/A. Material: Foetal membrane and primary amniotic epithelial cells. Methods: I F, q R T-q P C R, and Western blot. Marker: R A G E is present. 2. Spontaneous labour at term: Marker: increased R A G E in amnion and primary amniotic epithelial cells.

* 2018, Reference 79: Spontaneous labour at term. Sample 196 spontaneous vaginal delivery vs controls 49 elective and 42 emergency caesarean. Also 133 spontaneous and 99 induced labour vs 55 absent labour. Material: Cord venous blood. Method: E L I S A. Marker: increased H M G B 1.

* 2014, Reference 74: Endometrium in receptive phase. Control size 14 healthy women. Material: endometrial biopsy and uterine fluid. Methods: I H C, i T R A Q mass spectrometry, and Western blot. Markers: decreased H M G B 1 and decreased S 100 A 8.

Navigate back to Table 1..

### Figure 2. Long description

A schematic diagram divided into four vertical sections showing R A G E variants.

1. Top-Left Section: R A G E L I G A N D S. A cluster of yellow circles includes A beta, A G E s, s 1 0 0 P, M A C-1, H M G B 1, C 1 q, collagen I, I V, P S, and L P S. A green dashed arrow points from these ligands to the V domain of f l-R A G E.

2. Membrane-Bound R A G E Section: Shows f l-R A G E (full-length R A G E) spanning the cell membrane. It consists of an extracellular domain with three parts: a green V (variable domain), a blue C 1 (constant domain 1), and a larger blue C 2 (constant domain 2). Below these is a transmembrane domain and a red wavy cytosolic tail in the cytoplasm. A green arrow from the tail points to a box labeled ACTIVATION OF INTRACELLULAR SIGNALING.

3. s R A G E SOLUBLE RAGE Section: Shows two isoforms. c R A G E (cleaved) is shown being cut from the membrane by proteases A D A M 1 0 and M M P 9 (indicated by a scissor icon). e s R A G E (secreted) is shown as a splice variant. Both lack the transmembrane and cytosolic regions. Black arrows from these variants point toward the signaling box but are blocked by large red X marks.

4. Right Membrane-Bound Section: Shows d n-R A G E (dominant negative), which lacks the cytosolic tail, and N t-R A G E (N-truncated), which lacks the V domain. Black arrows from these to the signaling box are also blocked by red X marks.

5. Bottom Section (Intracellular): The ACTIVATION OF INTRACELLULAR SIGNALING box sends a green arrow into the nucleus to N F-kappa B. This triggers a green arrow pointing to a list: EXPRESSION OF PROINFLAMMATORY MOLECULES, EXPRESSION OF R A G E RECEPTORS, and EXPRESSION OF R A G E LIGANDS. A purple dashed arrow also points from the A G E R gene in the nucleus back up to the e s R A G E splice variant.

Navigate back to Figure 2..

### Figure 3. Long description

A flowchart diagram depicts the R A G E signaling pathway across three cellular compartments.

At the top in the extracellular space, A G Es bind to the V domains of a Y-shaped R A G E receptor, which consists of V, C 1, and C 2 domains. This binding triggers dimerization across the plasma membrane.

Below the membrane, the receptor tails activate two primary adaptor proteins: T I R A P on the left and D I A P H 1 on the right.

From T I R A P, the pathway branches into:

* N A D P H oxidase, which generates R O S.

* P I 3 K, which activates A K T and then G S K-3 beta.

From D I A P H 1, the pathway branches into:

* R A S, leading to M E K and E R K 1 / 2.

* R A C 1 / C D C 42, which splits into M K K 6 to P 38, and M K K 4 / 7 to S A P K / J N K.

* J A K, which activates S T A T 1.

At the bottom, these cascades converge on the nucleus. Transcription factors N F kappa B, E G R-1, A P-1, S T A T 3, and I S R E are activated and bind to a D N A strand labeled Pro-inflammatory Gene Transcription.

Two feedback loops are shown with dashed arrows exiting the nucleus:

1. Cytokine release leading to the expression of R A G E ligands.

2. A yellow solid line labeled Expression of R A G E and R A G E Upregulation, which points back to the extracellular receptor.

Navigate back to Figure 3..

### Table 2. Long description

The table consists of 8 columns: Year, Reference, Disease, Sample size, Control size, Material, Analysis method, and Marker.

* 2023 (Ref. 86): Endometriosis; Sample 24, Control 0; Follicular fluid; E L I S A; increased s R A G E.

* 2017 (Ref. 57): Endometriosis; Sample 84, Control 55; Eutopic and ectopic endometrium, menstrual blood, peripheral blood serum, peritoneal fluid; E L I S A, I H C; No difference with control.

* 2010 (Ref. 24): Endometriosis; Sample 28, Control 20; Endometriotic cyst, eutopic endometrium; q R T - P C R, Western blot; increased R A G E and increased E N - R A G E.

* 2010 (Ref. 88): Endometriosis; Sample 12, Control 10; Eutopic and ectopic endometrium; q R T - P C R, Western blot; increased R A G E and increased E N - R A G E.

* 2023 (Ref. 64): Endometriosis; Sample 51, Control 30; Peripheral blood plasma, peritoneal fluid; Multiplex inflammation panel; increased E N - R A G E in plasma.

* 2019 (Ref. 58): Endometriosis; Sample 30, Control 20; Ectopic endometrium, peripheral blood plasma; E L I S A, I H C; increased H M G B 1 in plasma and increased R A G E in highly fibrotic lesions.

* 2008 (Ref. 85): Endometriosis; Multiple samples (8 to 59), Multiple controls (4 to 78); Eutopic and ectopic endometrium, plasma, peritoneal and follicular fluid; E L I S A, I H C, q R T - P C R; increased s R A G E in follicular fluid.

* 2021 (Ref. 89): Endometriosis (ovarian endometrioma); Sample 10, Control 0; Ovarian cyst wall; q R T - P C R, Western blot; increased H M G B 1 and increased R A G E.

* 2024 (Ref. 65): Intrauterine adhesion – endometrial fibrosis; Sample N/A, Control N/A; Eutopic endometrium; I H C, q R T - P C R, Western blot; increased S 1 0 0 A 8, S 1 0 0 A 9, and R A G E.

* 2022 (Ref. 87): Intrauterine adhesion; Sample 6, Control 16; Scar tissue, myometrium; R N A - Seq; increased A G E - R A G E.

Navigate back to Table 2..

### Table 3. Long description

The table contains 8 columns: Year, Publication, Disease, Sample size, Control size, Material, Analysis method, and Marker.

* 2023 (Ref. 95): P C O S; Sample 21; Control 23; Granulosa lutein cells; q R T-P C R and R N A-Seq; increased S 1 0 0 A 9 and increased A G E-R A G E.

* 2020 (Ref. 96): P C O S; Sample 11; Control 10; Primary granulosa-lutein cells and ovarian tissue; I H C, Western blot, and q R T-P C R; increased R A G E and increased A G E s.

* 2007 (Ref. 25): P C O S; Sample 6; Control 6; Ovarian tissue biopsies; I H C; increased R A G E and increased A G E s.

* 2016 (Ref. 103): P C O S; Sample 555; Control 269; Peripheral blood; P C R-R F L P; R A G E polymorphisms -429T>C and -374T>A are not associated with P C O S.

* 2005 (Ref. 104): P C O S; Sample 29; Control 22; Peripheral blood (serum, P B M C s); E L I S A and flow cytometry; increased R A G E and increased A G E s.

* 2008 (Ref. 105): P C O S; Sample 100; Control 25; Peripheral blood (serum); E L I S A; increased A G E s.

* 2017 (Ref. 106): P C O S; Sample 148; Control 0; Peripheral blood (serum); E L I S A; increased A G E s and decreased s R A G E.

* 2023 (Ref. 94): P C O S; Sample 19; Control 26; Peripheral blood (serum) and follicular fluid; E L I S A; No difference in s R A G E F F and blood serum levels.

* 2024 (Ref. 107): P C O S; Sample 4; Control 4; Follicular fluid; Mass spectrometry; decreased R A G E 344-355.

* 2017 (Ref. 91): P C O S; Sample 12; Control 13; Follicular fluid; E L I S A; decreased s R A G E.

* 2017 (Ref. 92): P C O S; Sample 39; Control 35; Follicular fluid; E L I S A; decreased s R A G E.

* 2016 (Ref. 93): P C O S; Sample 39; Control 35; Follicular fluid; E L I S A; decreased s R A G E.

Navigate back to Table 3..

### Table 4. Long description

The table contains 17 rows of data across 8 columns.

* 2006 (Ref. 126): P E; Sample 10; Control 10; Serum and placental tissue; I H C, q R T-P C R, Western blot; increased R A G E and A G E s.

* 2007 (Ref. 127): P E; Sample 13; Control 12; Placental tissue; I H C, In situ hybridization; increased H M G B 1.

* 2011 (Ref. 119): Hypertensive disorders; Sample 60; Control 32; Placental tissue and plasma; E L I S A, I H C; increased R A G E and S 100 A 8 / S 100 A 9.

* 2011 (Ref. 120): P E and hypertension; Sample 13 and 38; Control 16 and 68; Blood, amniotic fluid, cord blood, placental tissue; E L I S A, q R T-P C R; increased s R A G E, es R A G E, and R A G E.

* 2015 (Ref. 26): P E; Sample 64; Control 61; Placental tissue; E L I S A, I F, I H C, Western blot; increased H M G B 1 and R A G E.

* 2016 (Ref. 115): P E and Severe P E; Sample 25 and 49; Control 110; Placental tissue; I H C, Western blot; increased H M G B 1 and significantly increased H M G B 1.

* 2016 (Ref. 116): P E; Sample N/A; Control N/A; Placental tissue; I H C, q R T-P C R, Western blot; increased R A G E protein.

* 2016 (Ref. 128): P E; Sample 21; Control 21; Trophoblast and blood; E L I S A, I H C, Western blot; increased H M G B 1, R A G E not affected.

* 2017 (Ref. 112): P E; Sample 18; Control 19; Placental tissue; I F, immunoprecipitation, q R T-P C R, Western blot; increased H M G B 1.

* 2017 (Ref. 121): P E; Sample 32; Control 30; Placental tissue, blood, umbilical blood; E L I S A, I H C, q R T-q P C R, Western blot; increased R A G E, A G E s, s R A G E, and decreased s R A G E in umbilical blood.

* 2021 (Ref. 113): P E; Sample 6; Control 6; Placental tissue; I F, Western blot; increased R A G E.

* 2023 (Ref. 48): P E; Sample 3; Control 3; Placental tissue; E L I S A, Western blot; No differences found.

* 2003 (Ref. 76): P E; Sample 11; Control 9; Myometrium and omental fat; I H C; significantly increased R A G E.

* 2010 (Ref. 123): P E; Sample 18; Control 79; Peripheral blood; E L I S A; increased s R A G E.

* 2011 (Ref. 124): P E; Sample 28; Control 87; Peripheral blood; E L I S A; increased s R A G E.

Navigate back to Table 4..

### Table 5. Long description

The table contains eight columns: Year, Publication, Disease, Sample size, Control size, Material, Analysis method, and Marker.

* 2021 (Ref. 113): Gestational diabetes mellitus; Sample 6, Control 6; Placental tissue; I F and Western blot; RAGE not affected.

* 2010 (Ref. 140): Gestational diabetes mellitus; Sample 150, Control 600; Peripheral blood; P C R-R F L P; RAGE polymorphisms -429T>C and -374T>A not associated.

* 2019 (Ref. 134): Gestational diabetes mellitus; Sample 12, Control 12; Foetal membrane, omental adipose tissue, peripheral blood; E L I S A and Western blot; increased H M G B 1 and increased RAGE.

* 2019 (Ref. 136): Gestational diabetes mellitus and vascular inflammation; N/A; HuVECs and plasma; Western blot; increased RAGE and increased A G Es.

* 2023 (Ref. 48): Gestational diabetes mellitus; Sample 3, Control 3; Placental tissue; E L I S A and Western blot; RAGE not affected.

* 2020 (Ref. 137): Gestational diabetes mellitus; N/A; Umbilical cord; I F and Western blot; increased RAGE and increased A G Es.

* 2016 (Ref. 116): Gestational diabetes mellitus; N/A; Placental tissue; I H C, q R T-q P C R, and Western blot; decreased RAGE m R N A and protein levels.

* 2021 (Ref. 133): Two entries for Impaired glucose tolerance (Sample 50, Control 71) and Gestational diabetes mellitus (Sample 59, Control 71); Peripheral blood; E L I S A; both show increased A G Es, RAGE, and C M L.

* 2009 (Ref. 138): Type I diabetes mellitus during pregnancy; Sample 29, Control 29; Peripheral blood; E L I S A; decreased s RAGE and increased A G Es.

* 2024 (Ref. 141): Hyperglycaemia; N/A; Human ovarian granulosa cell line; q R T-P C R; increased RAGE.

* 2018 (Ref. 135): Obesity; Sample 16, Control 17; Uterine fluid and endometrial biopsy; I H C and E L I S A; increased RAGE, A G Es, and C M L.

Navigate back to Table 5..

### Table 6. Long description

The table contains 22 entries detailing research on RAGE-related markers.

Key findings include:

* 2018 (Ref. 145): In p P R O M, placental biopsy and blood showed increased H M G B 1 and R A G E via E L I S A, I F, q R T-P C R, and Western blot.

* 2015 (Ref. 146): Peripheral blood showed increased es R A G E in p P R O M and preterm rupture at term.

* 2021 (Ref. 147): Amniotic fluid showed increased E N-R A G E in threatened preterm labour.

* 2009 (Ref. 161): Amniotic fluid and cord blood showed decreased s R A G E in severe chorioamnionitis.

* 2008 (Ref. 152) and 2012 (Ref. 162): Chorioamnionitis studies showed increased es R A G E, s R A G E, and H M G B 1, but 2012 noted decreased s R A G E.

* 2022 (Ref. 150) and 2016 (Ref. 149): Chorioamnionitis showed increased E N-R A G E, S 100 A 8/A 9, and H M G B 1.

* 2007 (Ref. 151): Positive and negative culture chorioamnionitis both showed increased E N-R A G E and decreased s R A G E.

* Spontaneous preterm labour studies (2010-2023): Findings include increased H M G B 1, E N-R A G E, and S 100 A 8/A 9, while s R A G E was often decreased (Ref. 123, 157, 155).

* 2022 (Ref. 159): Zika virus infection showed increased R A G E in placental tissue but decreased R A G E in trophoblast cells.

Analysis methods frequently include E L I S A, Western blot, and I F. Materials sampled range from amniotic fluid and peripheral blood to placental biopsies and foetal membranes.

Navigate back to Table 6..

### Table 7. Long description

The table consists of eight columns: Year, Publication, Disease, Sample size, Control size, Material, Analysis method, and Marker.

* Row 1: 2022, Ref. 166, Recurrent spontaneous abortion, sample size 31, control size 26, Trophoblast tissue, Flow cytometry, q R T - P C R, and Western blot, decreased RAGE.

* Row 2: 2017, Ref. 167, Recurrent spontaneous abortion, sample size 60, control size 20, Peripheral blood, E L I S A and Protein antibody microarray, decreased RAGE.

* Row 3: 2014, Ref. 170, Recurrent spontaneous abortion, sample size 63, control size 30, Peripheral blood, E L I S A, increased sRAGE.

* Row 4: 2020, Ref. 168, Unexplained recurrent spontaneous abortion, sample size 55, control size 55, Villus and decidual tissue and peripheral blood, I F, I H C, E L I S A, and Western blot, increased H M G B 1 and increased RAGE.

* Row 5: 2021, Ref. 169, Unexplained Recurrent Spontaneous Abortion, sample size 75, control size 75, Decidual tissue, Western blot, increased H M G B 1.

Navigate back to Table 7..

### Table 8. Long description

The table consists of eight columns: Year, Publication, Disease, Sample size, Control size, Material, Analysis method, and Marker.

* 2023, Reference 48: Intrauterine growth restriction I U G R, sample size 3, control size 3, material placenta, analysis by E L I S A and Western blot, marker R A G E not effected.

* 2012, Reference 122: I U G R, sample size 22, control size 120, material peripheral blood, analysis by E L I S A and polymorphism analysis, marker no differences.

* 2016, Reference 116: I U G R, sample size N/A, control size N/A, material placenta, analysis by I H C, q R T-q P C R, and Western blot, marker decreased R A G E protein.

* 2013, Reference 172: Infant morbidities, sample size 130, control size N/A, material infant peripheral blood, analysis by E L I S A, marker increased s R A G E and increased S 100 B in maternal chorioamnionitis, and decreased s R A G E in sepsis, respiratory failure, and maternal preeclampsia.

* 2017, Reference 173: Brain injury in preterm infants, sample size 44, control size 67, material foetal membranes and umbilical cord blood, analysis by E L I S A and I H C, marker increased H M G B 1, increased R A G E, and decreased s R A G E.

* 2012, Reference 122: Intrahepatic pregnancy cholestasis, sample size 14, control size 120, material peripheral blood, analysis by E L I S A and polymorphism analysis, marker no differences.

* 2020, Reference 174: Intrauterine H B V infection, sample size 16, control size 26, material neonate peripheral blood and placenta, analysis by I H C, mass spectroscopy proteomics, and Western blot, marker increased S 100 A 8, increased S 100 A 9, and increased S 100 A 12.

Navigate back to Table 8..

### Table 9. Long description

The table contains eight columns: Year, Publication, Disease, Sample size, Control size, Material, Analysis method, and Marker.

* 2019, Ref 175: Antiphospholipid syndrome (A P S), 60 samples, 30 controls, peripheral blood serum, Western blot, increased H M G B 1 and increased s R A G E.

* 2017, Ref 176: Antiphospholipid syndrome (A P S), 30 samples, 30 controls, peripheral blood serum, Western blot, increased H M G B 1 and increased s R A G E.

* 2018, Ref 177: Pelvic organ prolapse, 20 samples, 10 controls, vaginal tissue, I H C and Western blot, increased A G Es.

* 2017, Ref 178: Pelvic organ prolapse, 3 samples, 3 controls, vaginal tissue, q R T - P C R and Western blot, increased R A G E.

* 2015, Ref 179: Pelvic organ prolapse, 44 samples, 46 controls, vaginal tissue, I H C, genotyping, and Western blot, increased A G Es.

* 2022, Ref 180: Acute cervical insufficiency, 50 samples, 0 controls, amniotic fluid, E L I S A, increased R A G E.

* 2022, Ref 181: Cervical insufficiency, 80 samples, 49 controls, amniotic fluid, Antibody-based protein microarray and E L I S A, increased E N - R A G E and increased S 100 A 8 / A 9.

* 2014, Ref 185: Ovarian ageing, N / A samples, N / A controls, primary ovarian granulosa-lutein cells and monocytes, I F and flow cytometry, increased A G Es and increased R A G E.

* 2014, Ref 183: Diminished ovarian reserve, 33 samples, 31 controls, follicular fluid and peripheral blood serum, E L I S A, decreased s R A G E in follicular fluid.

* 2017, Ref 184: Infertility and advanced maternal age (A M A), 62 young infertile and 62 A M A women, 0 controls, follicular fluid, E L I S A, decreased s R A G E.

* 2013, Ref 186: I V F embryo quality, 26 poor-quality and 19 high-quality embryos, follicular fluid, Luminex x M A P, decreased s R A G E in high-quality embryos.

* 2010, Ref 182: Ageing, 114 elder women and 77 young women, peripheral blood plasma and follicular fluid, E L I S A, decreased s R A G E in blood plasma.

Navigate back to Table 9..

### Table 10. Long description

The table contains 8 columns: Year, Publication, Disease, Sample size, Control size, Material, Analysis method, and Marker.

Key findings include:

* 2013 (Ref. 194): Chronic cervicitis, C I N, and Squamous cervical cancer show progressive increases in S 1 0 0 A 9 and R A G E (from increased to substantially increased) using I H C on tissue samples.

* 2021 (Ref. 205): Chronic cervicitis in H C E cell lines shows increased H M G B 1 and R A G E via Western blot.

* 2017 (Ref. 193 and 192): C I N and cervical cancer show increased S 1 0 0 A 7 and H M G B 1 via I H C.

* 2021 (Ref. 191): Cervical cancer shows increased R A G E in tumor tissue via Western blot, I H C, and I F.

* 2018 and 2012 (Ref. 206, 195): Cervical cancer studies on peripheral blood show decreased G T / T T genotype and decreased s R A G E.

* 2013 (Ref. 196): Epithelial ovarian cancer study links 82 G to S polymorphism of R A G E gene to susceptibility.

* 2017 (Ref. 198): Ovarian cancer in A 2 7 8 0 cell lines and G E O database shows increased S 1 0 0 B and R A G E.

* 2023 (Ref. 27): Ovarian cancer serum shows significantly increased H M G B 1 and significantly decreased s R A G E via E L I S A.

* 2015 (Ref. 197): Serous ovarian carcinoma shows increased R A G E via I H C.

* 2016 (Ref. 200): Endometrial cancer shows increased R A G E in tissue samples via I H C.

Navigate back to Table 10..

### Table 11. Long description

The table contains nine columns: Year, Publication, Species, Disease, Sample size, Control size, Material, Analysis method, and Marker.

Key findings across the rows include:

* Gestational diabetes mellitus: Multiple studies in rats and rabbits (2014 to 2024) show increased concentrations of A G E s and R A G E in peripheral blood, placenta, and fetal tissues using E L I S A, q R T - P C R, and Western blot.

* P C O S: Studies in mice (2020 to 2023) show increased R A G E in peripheral blood and ovarian tissue, though one study noted decreased R A G E in the uterus despite increased M G - A G E s.

* Intra-amniotic infection: A 2021 pig study found decreased R A G E and H M G B 1 in the amniotic membrane but increased H M G B 1 and decreased s R A G E in amniotic fluid.

* Prenatal cleft palate: Mouse studies (2017, 2020) show conflicting s R A G E levels (one increased, one decreased) in maternal blood and embryos.

* Other conditions: Increased R A G E is noted in prenatal lung development after nicotine exposure, intrauterine growth restriction from second-hand smoke, and diabetic embryopathy. A 2024 study on endometritis in mares showed no difference in markers.

* Analysis methods frequently cited include E L I S A, q R T - P C R, I H C, and Western blot. Species studied include rats, mice, rabbits, pigs, mares, and bovine models.

Navigate back to Table 11..

### Figure 4. Long description

The diagram is divided into two rows of three panels each.

Top Row: Extracellular Inhibition

* Panel 1: A SMALL MOLECULE INHIBITOR (red sphere) binds to the distal end of the fl-R A G E receptor, blocking R A G E LIGANDS (orange spheres) from attaching. A red X over the intracellular tail indicates SIGNAL OFF.

* Panel 2: ANTI-R A G E ANTIBODIES (Y-shaped proteins) bind to the fl-R A G E receptor, preventing ligand binding. A red X indicates SIGNAL OFF.

* Panel 3: esRAGE ACTS AS DECOY RECEPTOR. Soluble receptors (green and blue segments) sequester R A G E LIGANDS in the extracellular space. A green checkmark shows successful sequestration, while a red X on the membrane-bound fl-R A G E indicates SIGNAL OFF.

Bottom Row: Intracellular and Precursor Inhibition

* Panel 4: An AGE FORMATION INHIBITOR (pink sphere with a T-bar) blocks the conversion of AGE PRECURSOR into A G E. This prevents ligands from reaching the fl-R A G E receptor, resulting in SIGNAL OFF.

* Panel 5: s i R N A mediated degradation. Double-stranded s i R N A combines with R I S C (tan cloud). The complex binds to R A G E m R N A. A pair of scissors icon represents the cleavage of the m R N A, preventing protein synthesis.

* Panel 6: A S O mediated inhibition. In the top half, an A S O (green strand) binds to R A G E m R N A, and R N Ase H (purple enzyme) degrades the m R N A. In the bottom half, an A S O blocks the TRANSLATION START SITE, preventing a RIBOSOME (light green) from producing R A G E PROTEIN, indicated by a red X over a protein chain.

Navigate back to Figure 4..

### Table 12. Long description

The table contains 42 rows of data across 7 columns.

* Column 1: Year (ranging from 2014 to 2025).

* Column 2: Publication (Reference numbers).

* Column 3: Condition (P C O S, Premature ovarian failure, Ovarian dysfunction, Endometriosis, Pelvic inflammatory disease, Pelvic organ prolapse, Cervical inflammation, Gestational diabetes mellitus, Preeclampsia, Spontaneous abortion, and various cancers).

* Column 4: Model (Bioinformatic analysis, mouse/rat models, or specific cell lines like human ovarian granulosa cells).

* Column 5: Medication (Herbal decoctions like Cangfu Daotan, proteins like P E D F, drugs like Metformin or Aspirin, and genetic modifications like s i R N A R A G E silencing).

* Column 6: Type of medication (Herbal medicine, Recombinant protein, Non-specific R A G E modulator, R A G E inhibitor, or Genetic modifications).

* Column 7: Outcome (Primarily showing a decrease in A G E-R A G E pathways, oxidative stress, and inflammation).

Key trends include:

* P C O S treatments (2018-2023) using herbal medicines like Leonuri Herba and Erxian Decoction consistently result in a decreased A G E-R A G E pathway.

* Gestational diabetes mellitus studies (2014-2024) using Crocetin, Baicalin, or R A G E knockout models show decreased fetal developmental defects and reduced oxidative stress.

* Cancer studies (Endometrial, Ovarian, Cervical, and Breast) utilize R A G E inhibitors like F P S-Z M 1 or genetic silencing to decrease cancer cell proliferation and migration potential.

Navigate back to Table 12..

### Figure 5. Long description

The diagram is divided into two main sections.

Left Section: Physiology Moderate Activity.

- Rupture of Foetal Membranes: R A G E up arrow, H M G B 1 up arrow.

- Decidualization and Blastocyst Implantation: R A G E up arrow.

- Regulation of Endometrial Receptivity: R A G E down arrow.

- Physiological Aging: A G E S up arrow, R A G E up arrow, s R A G E down arrow.

- Foetal Development: Illustrated by a sequence of growing foetuses.

Right Section: Pathophysiology Significant Overactivation.

- Pregnancy Disorders: Hypertension in Pregnancy (R A G E up, H M G B 1 up, S 100 A 12 up) and Gestational Diabetes Mellitus (Glucose up, R A G E up, A G E S up, s R A G E down).

- Foetal Development Defects: R A G E up, H M G B 1 up, s R A G E down.

- Premature Birth: H M G B 1 up, S 100 A 12 up, s R A G E down.

- Pregnancy Loss: R A G E up, H M G B 1 up.

- Endometriosis: R A G E up, H M G B 1 up, S 100 A 12 up.

- P C O S: A G E S up, R A G E up, s R A G E down.

- Gynecological Malignances: Ovarian Cancer (R A G E up, s R A G E down, H M G B 1 up, S 100 B up), Cervical Cancer (R A G E up, S 100 A 9 up, H M G B 1 up), and Endometrial Cancer (R A G E up, S 100 A 8 up, H M G B 1 down).

Navigate back to Figure 5..
